# Metabolic dysregulation in Alzheimer's disease: A brain metabolomics approach

**DOI:** 10.1002/alz.70528

**Published:** 2025-09-11

**Authors:** Anke Hüls, Youran Tan, Emma Casey, Zhenjiang Li, Marla Gearing, Allan I. Levey, James J. Lah, Aliza P. Wingo, Dean P. Jones, Douglas I. Walker, Thomas S. Wingo, Donghai Liang

**Affiliations:** ^1^ Department of Epidemiology Rollins School of Public Health Emory University Atlanta Georgia USA; ^2^ Gangarosa Department of Environmental Health Rollins School of Public Health Emory University Atlanta Georgia USA; ^3^ Department of Biostatistics and Bioinformatics Rollins School of Public Health Emory University Atlanta Georgia USA; ^4^ Goizueta Alzheimer's Disease Research Center School of Medicine Emory University Atlanta Georgia USA; ^5^ Department of Pathology and Laboratory Medicine Emory University Atlanta Georgia USA; ^6^ Department of Neurology Emory University School of Medicine Atlanta Georgia USA; ^7^ Department of Psychiatry University of California, Davis Sacramento California USA; ^8^ Division of Mental Health Northern California VA Sacramento California USA; ^9^ Department of Medicine Emory University Atlanta Georgia USA; ^10^ Department of Neurology University of California, Davis Sacramento California USA; ^11^ Alzheimer's Disease Research Center University of California, Davis Sacramento California USA

**Keywords:** Alzheimer's disease, amino acid metabolism, carbohydrate metabolism, high‐resolution metabolomics, lipid metabolism, metabolism of cofactors and vitamins, neuropathology, nucleotide metabolism, untargeted metabolomics

## Abstract

**INTRODUCTION:**

This study aimed to identify specific biological pathways and molecules involved in Alzheimer's disease (AD) neuropathology.

**METHODS:**

We conducted cutting‐edge high‐resolution metabolomics profiling of 162 human frontal cortex samples from the Emory Alzheimer's Disease Research Center (ADRC) brain bank with comprehensive neuropathological evaluations.

**RESULTS:**

We identified 155 unique metabolic features and 36 pathways associated with three well‐established AD neuropathology markers. Of these, 18 novel metabolites were confirmed with level 1 evidence, implicating their involvement in amino acid metabolism, lipid metabolism, carbohydrate metabolism, nucleotide metabolism, and metabolism of cofactors and vitamins in AD neuropathology. Genetic variability influenced these associations, with non‐carriers of the apolipoprotein E (*APOE*) ε4 allele showing stronger perturbations in metabolites including glucose and adenosine 5′‐diphosphoribose.

**DISCUSSION:**

This study demonstrates the potential of high‐resolution metabolomic profiling in brain tissues to elucidate molecular mechanisms underlying AD pathology. Our findings provide critical insights into metabolic dysregulation in AD and its interplay with genetic factors.

**Highlights:**

- This is one of the largest untargeted metabolomics studies of human brain tissue.
- 155 metabolic features, and 36 metabolic pathways were linked to Alzheimer's disease (AD) neuropathology.
- Of these, 18 unique metabolites were confirmed with level 1 evidence.
- Glucose and adenosine 5′‐diphosphoribose identified as key metabolic alterations in AD.

## 1 BACKGROUND

Alzheimer's disease (AD) and related dementias are the 6th leading cause of death affecting over six million people in the United States, and the number is projected to grow to 13.8 million by 2060.^1^, ^2^ AD is the most common cause of dementia and its hallmark pathologies include accumulation of β‐amyloid (Aβ plaques) outside neurons and aggregation of hyperphosphorylated tau protein (neurofibrillary tangle [NFT]) inside neurons in the brain.^3^ Individuals living with AD experience cognitive impairment that compromises their quality of life and often need long‐term day‐to‐day support and care.^1^ Therefore, it is critical to develop effective approaches to reduce disease burden and associated costs.

Much effort is currently focused on developing therapies to block the progression of AD, including drugs targeting key molecular pathways involved in disease pathogenesis.^4^ To develop and optimize disease‐modifying treatments, it is critical to understand the underlying biological mechanisms, including the production and aggregation of Aβ plaques, the formation of NFTs composed of hyperphosphorylated tau, and other interconnected processes such as neuroinflammation, oxidative stress, and synaptic dysfunction.^5^, ^6^ Metabolomics is an emerging omics technology capable of characterizing thousands of small molecules and perturbations of endogenous biological pathways in tissues or biofluids, and metabolic dysfunction is a known core feature of AD.^7^ Thus, understanding the brain metabolome in relation to AD could point towards discovery of novel targets for intervention and biomarkers reflecting environmentally associated changes in the brain.

The application of metabolomics in AD etiology remains nascent,^8^, ^9^ and most previous brain tissue‐based metabolomics studies of AD have utilized targeted approaches. With these approaches, metabolites belonging to the sphingolipid and glycerophospholipid classes,^10^, ^11^ acylcarnitine and biogenic amine,^11^ and changes in several neurotransmitter‐related^12^ and amino acid metabolites such as methionine, alanine, aspartate, glutamine, and arginine^11^, ^12^, ^13^ have been identified in association with AD progression, the severity of AD pathology in the brain and AD diagnoses. Despite these promising initial evidences, targeted metabolomics has limited ability to comprehensively detect and characterize novel targets for future intervention. In particular, these targeted metabolomics investigations are typically capable of profiling only a few hundred metabolites, which may miss novel or low‐abundance molecular signatures relevant to AD pathology.

In contrast, untargeted metabolomics, which aims for universal and unbiased profiling of the metabolome, provides a more holistic measure of molecular signatures of exposures and diseases. While few untargeted studies have investigated the brain metabolome and AD etiology, these initial applications have shown great promise in identifying metabolites and related pathways associated with AD neuropathology^14^ and AD diagnoses,^15^, ^16^, ^17^ including many novel pathways that were not the focus of previous targeted metabolomics studies. Recent work has also uncovered region‐specific metabolic alterations in AD^18^ and disease‐specific traits of AD and tauopathies,^19^ providing a broader view of metabolic dysregulation in neurodegeneration. For example, Batre et al. analyzed dorsolateral prefrontal cortex samples and found disruptions in cholesterol metabolism, steroid biosynthesis, and neuroinflammatory pathways.^20^ However, previous studies typically detected fewer than 1,000 metabolic signatures using a commercial untargeted platform and often focused on case—control comparisons, with limited investigation of specific neuropathological markers such as Braak stage or Consortium to Establish a Registry for Alzheimer's Disease (CERAD) score.^19^, ^21^, ^22^, ^23^, ^24^ Thus, a larger systematic investigation with more comprehensive metabolomics feature coverage is warranted to replicate and validate the initial findings, as well as to identify additional metabolites and pathways in the brain metabolome that can provide novel mechanistic insights on AD neuropathology.

To address these knowledge gaps, we leveraged data from the Emory Goizueta Alzheimer's Disease Research Center (ADRC) to assess untargeted metabolomics in prefrontal cortex tissues of 162 brain donors with different stages of AD neuropathology. We hypothesized that the use of this large dataset, together with an innovative high‐resolution metabolomics approach that can simultaneously assess >40K unique metabolic signals, would deepen our knowledge about the various metabolic pathways involved in the development of AD and could consequently help to identify novel targets for intervention.

**RESEARCH IN CONTEXT**

1. **Systematic review**: Alzheimer's disease (AD) is a complex neurodegenerative disorder characterized by progressive cognitive decline and neuropathological hallmarks. Despite extensive research, the molecular mechanisms linking metabolic dysregulation to AD neuropathology remain poorly understood. Metabolomics, a powerful tool for profiling small molecules, provides an opportunity to identify metabolic signatures and pathways implicated in disease progression. To address this gap, we conducted one of the largest high‐resolution brain metabolomics studies to date, profiling metabolic perturbations associated with AD neuropathology.
2. **Interpretation**: Our findings demonstrate the potential of high‐resolution metabolomic profiling in brain tissues to elucidate molecular mechanisms underlying AD pathology. For the first time, we link 18 novel metabolites in the human brain with gold standard evidence to AD neuropathology, including glucose and adenosine 5′‐diphosphoribose, demonstrating important metabolic alterations in AD.
3. **Future directions**: Future longitudinal studies are needed to validate these findings and explore the functional significance of the identified metabolites in AD progression.

## 2 METHODS

### 2.1 Study population

Brain tissue donors were recruited by the Emory ADRC. The Emory ADRC maintains a brain bank to facilitate AD research. As described previously,^25^, ^26^, ^27^ the majority of donors were research participants, and others were in the Emory ADRC clinical core or patients treated by Emory physicians and diagnosed clinically with Alzheimer's disease (biomarker defined) or probable Alzheimer's disease. There were 1011 donors in the brain bank by the third quarter of 2020. Among these donors, untargeted metabolomics was conducted in 162 available samples from the donors who were deceased after 2007 at the age of 55 years or older with no missing values in neuropathology outcomes (i.e., Braak stage, CERAD score, and the combined Alzheimer's disease neuropathologic change [ABC score]) and key covariates including age at death, calendar year of death, race, sex, educational attainment, and apolipoprotein E (*APOE)* genotype. Written informed consent was obtained for all donors. Samples were obtained using research protocols approved by the Emory University Institutional Review Board.

### 2.2 Assessment of AD neuropathology

As described previously,^25^, ^26^, ^27^ the Emory ADRC performed thorough neuropathologic evaluations on the brains of all donors using established comprehensive research evaluations and diagnostic criteria.^28^ All neuropathologic examinations included assessment of the severity of Alzheimer's disease‐related neuropathologic changes using a variety of histologic stains and immunohistochemical preparations, as well as semi‐quantitative scoring of multiple neuropathologic changes in numerous brain regions by experienced neuropathologists using published criteria. The measures of Alzheimer's disease severity used in this project were the Braak stage,^29^ the CERAD score,^30^ and the ABC score.^31^ Braak stage divides NFTs into seven levels of severity (Stage 0–VI), and a higher stage indicates a wider distribution of NFTs in the brain. CERAD score describes the prevalence of neuritic plaques in the neocortex, with four levels ranging from no neuritic plaques to frequent. ABC score combines the former two with the distribution of amyloid plaques in the brain (Thal score,^32^ ranging from 0 to 5) to yield one of four levels of Alzheimer's disease neuropathologic changes: none, low, intermediate, or high.

### 2.3 High‐resolution metabolomics

High‐resolution metabolic profiling was conducted on prefrontal cortex tissue using previously established protocols.^33^, ^34^, ^35^, ^36^ Each sample was analyzed in triplicate using liquid chromatography with high‐resolution mass spectrometry (LC‐HRMS) techniques (Dionex Ultimate 3000, Thermo Scientific Q Exactive HF). Two chromatography analytical columns were applied; hydrophilic interaction liquid chromatography (HILIC) with positive electrospray ionization (ESI), and reverse phase (C18) chromatography with negative ESI, as having data from both columns can enhance the coverage of metabolic feature detection to provide a more comprehensive profile of metabolism. Typically, highly polar metabolites are better separated by the HILIC column, and less polar or long chain metabolites, phospholipids, or polyphenolic compounds can be captured and separated by C18 column.^37^ Each detected signal was characterized by an accurate mass (5 ppm) measure of the mass‐to‐charge ratio (*m/z*), associated retention time (RT) for elution from the chromatography column and integrated ion intensity for relative quantification. After the analytical run, raw instrument files were converted to .mzML format using ProteoWizard and signals were extracted using apLCMS with modifications by xMSanalyzer, which performed peak detection, *m/z* and RT alignment, feature quantification, batch correction, and data quality filtering.^38^, ^39^ To filter out the noise signals and optimize the metabolomics data quality, only metabolic features detected in >15% of brain tissues with a median coefficient of variation (CV) among technical replicates <30% and Pearson correlation *ρ* > 0.7 were included in further analyses. The resulting analytic data contained individual features defined by *m/z*, RT, and ion intensities. Then, we averaged the replicate samples that had at least one non‐zero intensity and performed a log 2 transformation to normalize the metabolomics data for statistical analysis.

### 2.4 Statistical analysis

The associations of metabolic features and AD neuropathology markers were assessed following an untargeted metabolome‐wide association study (MWAS) workflow. MWAS models were conducted using ordinal logistic regression (OLR) to identify significant features associated with the outcome of interest, controlling for age at death, race, sex, educational attainment, post‐mortem interval (PMI), and *APOE* genotype using the following form:

$$\begin{array}{ccc} In\left(\frac{P\left(D\;\geq g\;|X\right)\;}{P\left(D\;<g\;|X\right)\;}\right) & = & \alpha_{gj}+\beta_{1j}\;log_{2\;}\left(\mathrm{Intensity}\right)+\beta_{2j}\;\mathrm{Age}\;\mathrm{at}\;\mathrm{death}\\ & & +\;\beta_{3j}\;\mathrm{Race}+\beta_{4j}\mathrm{Sex}+\beta_{5j}\;\mathrm{Education}\\ & & +\;\beta_{6j}\;\mathrm{postmortem}\;\mathrm{interval}+\beta_{7j}\;APOE \end{array}$$

where intensity denotes the intensity of each metabolic feature *j*. *D* represents the three different AD neuropathology markers, respectively (ABC, Braak Stage, CERAD), and *g* represents the ordinal categories of each outcome. $\alpha_{g}$ represents the intercepts for different categories, $\beta_{1-7}$ are coefficients corresponding to each predictor, and single odds ratio was reported for different ordinal categories. The race variable was binary as the current sample only contained White and Black participants. The educational attainment referred to the highest level of education that the subjects had completed, which was estimated based on number of years of education completed and classified into high school or less, college degree, and graduate degree. PMI refers to time (hours) between death and brain removal was treated as continuous variable. The *APOE ε4* allele is a well‐known risk factor of developing Alzheimer's disease, and the current analysis considered the dosage of *ε4* allele (0, 1, and 2). Analyses were conducted separately for each column (HILIC positive ESI and C18 negative ESI) and each AD neuropathology marker (ABC, Braak Stage, CERAD).

Proportional odds (PO) assumption was examined for each metabolic feature using the *Score Test*.^40^ Only features that met the PO assumptions (*p* value > 0.05) were considered in the final analyses. We used the Benjamini–Hochberg (false discovery rate [FDR]) method for multiple comparison correction. All analyses were conducted in R (version 4.2.0).

### 2.5 Pathway enrichment analysis and chemical annotation

To predict the functional activity of metabolic features from LC‐HRMS output, we conducted pathway enrichment and chemical annotation analyses. Pathway enrichment was performed using the R package *metapone* with a unadjusted *p* value of 0.05 as cutoff.^41^ *Metapone* is a novel bioinformatic platform to predict functional biological activities of untargeted metabolomic data, without relying on identified or validated metabolites for pathway mapping.^41^ To minimize the chance of false positive discovery, we only included biological pathways associated with AD neuropathology markers identified by *metapone* with adjusted *p* value less than 0.05 and at least four significant metabolites enriched that were matched with known compounds by *m/z*.

To reduce false positive discovery, for each significant metabolic feature significantly associated with AD neuropathology markers (*p* value < 0.05) and also enriched in a relevant pathway, we screened on their retention time, isotope patterns, and spectrum peak quality by visually examining the extracted ion chromatographs (EICs) to differentiate the true peak from the noise (exhibiting clear Gaussian peak shapes and signal‐to‐noise ratios above 3:1). The features passing the examination were annotated and confirmed using the Metabolomics Standards Initiative criteria.^42^, ^43^ Specifically, to further minimize false positive matches, only features whose *m/z* (±10 ppm difference) and retention time (±10 s) matched the authentic compounds analyzed under identical experimental conditions were assigned with Level‐1 confidence.[Table alz70528-tbl-0001]

### 2.6 Sensitivity analyses

Several sensitivity analyses were conducted to assess the robustness of results. First, we replaced OLR with multiple linear regression (MLR), which considered the neuropathology markers as continuous variables. Second, we additionally controlled for cell type heterogeneity among samples by controlling for proportions of neuronal cells estimated from the matched DNA methylation samples (see Ref. 26 for details). Third, we adjusted for the first three principal components from the metabolic features in the regression model to control for additional technical variation that had not been adjusted during the batch correction.^38^, ^39^ Fourth, to evaluate the effect of using different cut‐offs of *q*‐ and *p*‐values for the significance in pathway enrichment analysis, we performed sensitivity analyses using *q* value < 0.2 and raw *p* value < 0.01. Finally, to better understand how the associations between the significant metabolic features and AD neuropathology markers were influenced by *APOE* ε4 genotype, we included a multiplicative interaction term between the significant metabolic features that were confirmed with level 1 evidence^42^ and *APOE* genotype (presence or absence of ε4 allele) to test for effect modification and presented the stratified effect estimates derived from that interaction model.

## 3 RESULTS

### 3.1 Study population

Mean age at death for the 162 donors included in this analysis was 76.2 years (SD: 9.3 years). The majority of donors were White (89.5%), male (56.2%), and had a college degree or higher level of education (77.7%). In our sample, 43.8% had one *APOE ε4* allele and 13.0% had two copies. Almost half of the participants were classified as having the highest Braak stage (46.3%), and over half were in the highest CERAD (69.8%) and ABC (58.6%) categories (Table 1). Based on the ABC score, 72.7% of the donors had pathology‐confirmed AD (classified as intermediate or high ABC scores). Based on clinical diagnosis, most participants were diagnosed with Alzheimer's disease (52.5%), followed by frontotemporal dementia (13%), and Amyotrophic lateral sclerosis (9.3%).

**TABLE 1 alz70528-tbl-0001:** Characteristics of study population (*N* = 162).

Parameter	Overall (*N* = 162)
Age at death	
Mean (SD)	76.22 (9.29)
Race	
Black	17 (10.5%)
White	145 (89.5%)
Sex	
Female	71 (43.8%)
Male	91 (56.2%)
Education	
College degree	78 (48.1%)
Graduate degree	48 (29.6%)
High school or less	36 (22.2%)
Post‐mortem interval (PMI)^a^	
Mean (SD)	11.75 (9.61)
Apoliprotein E genotype	
No E4	70 (43.2%)
One E4	71 (43.8%)
Two E4	21 (13.0%)
Neuronal cell%^a^	
Mean (SD)	0.32 (0.08)
ABC	
Not	15 (9.3%)
Low	29 (17.9%)
Intermediate	23 (14.2%)
High	95 (58.6%)
Braak stage	
Stage 1	16 (9.9%)
Stage 2	11 (6.8%)
Stage 3	20 (12.3%)
Stage 4	18 (11.1%)
Stage 5	22 (13.6%)
Stage 6	75 (46.3%)
CERAD	
No	35 (21.6%)
Sparse	4 (2.5%)
Moderate	10 (6.2%)
Frequent	113 (69.8%)
Primary clinical diagnosis	
AD	85 (52.5%)
ALS	15 (9.3%)
CBD	4 (2.4%)
Dementia	2 (1.2%)
DLB	11 (7.0%)
FTD	21 (13.0%)
Leukoencephalopathy	1 (0.6%)
MCI	6 (3.7%)
PD	2 (1.2%)
PDD	3 (1.8%)
PPA	1 (0.6%)
PSP	4 (2.4%)
Control	7 (4.3%)

Abbreviations: ABC, NIA‐AA Alzheimer's disease neuropathologic change (ADNC); AD, Alzheimer's disease; ALS, amyotrophic lateral sclerosis; BRAAK, Braak stage for neurofibrillary degeneration; CBD, corticobasal degeneration; CERAD, Consortium to Establish a Registry for Alzheimer's Disease score for density of neocortical neuritic plaques; DLB, dementia with Lewy bodies; FTD, frontotemporal dementia; MCI, mild cognitive impairment; PD, Parkinson's disease; PDD, Parkinson's disease dementia; PPA, primary progressive aphasia; PSP, progressive supranuclear palsy.

^a^ Three subjects were missing on post‐mortem interval (PMI) and two subjects were missing on cell type.

### 3.2 MWAS of AD neuropathology

After data quality assurance and quality control, we extracted 20,051 and 15,927 metabolic features from frontal cortex samples using HILIC positive ESI and C18 negative ESI, respectively (Table S1). Of these, 590 and 529 metabolites were confirmed with Level 1 evidence in HILIC positive ESI and C18 negative ESI columns, respectively (Table S1).

In total, 155 metabolic features were significantly associated with AD neuropathology markers after adjusting for age at death, race, sex, educational attainment, PMI, and *APOE* genotype and multiple testing (*q* values < 0.05), including 26, 17, and 20 metabolic features in HILIC column, and 64, 12, and 16 metabolic features in C18 column for ABC, Braak Stage, CERAD, respectively (Table 2). When using *q* values < 0.2 as a threshold for significance, these numbers increased to 45, 35, and 23 metabolic features in the HILIC column, and 765, 97, and 20 metabolic features in the C18 column, in associations with ABC, Braak Stage, and CERAD, respectively. Generally, more significant metabolic features were found in the hydrophobic C18 column compared to the hydrophilic HILIC column after FDR correction.

**TABLE 2:** Number of metabolic features associated with three Alzheimer's disease neuropathology markers.

	HILIC ESI+ (No. metabolic features = 20,051)				C18 ESI– (No. metabolic features = 15,927)			
AD neuropathology marker	FDR *q* value < 0.05	FDR *q* value < 0.2	Raw *p* value < 0.01	Raw *p* value < 0.05	FDR *q* value < 0.05	FDR *q* value < 0.2	Raw *p* value < 0.01	Raw *p* value < 0.05
ABC	26	45	457	1712	64	765	756	2115
Braak stage	17	35	174	606	12	97	92	305
CERAD	20	23	191	1001	16	20	193	890

*Note*: FDR indicates Benjamini–Hochberg procedure for false discovery rate correction of multiple comparisons. Associations were estimated using an ordinal logistic regression model, adjusted for donors’ race, sex, education, age at death, PMI, and apoliprotein E genotype.

Abbreviations: ABC, NIA‐AA Alzheimer's disease neuropathologic change (ADNC); AD, Alzheimer's disease; BRAAK, Braak stage for neurofibrillary degeneration; CERAD, Consortium to Establish a Registry for Alzheimer's Disease score for density of neocortical neuritic plaque; ESI, electrospray ionization; FDR, false discovery rate; HILIC, hydrophilic interaction liquid chromatography;.

Comparing the significant metabolic features (*q* value < 0.2) that overlapped between the three neuropathology scores showed that most metabolic features were exclusively associated with ABC compared to Braak Stage and CERAD in both HILIC column (*q* value < 0.2: 31%) and C18 column (*q* value < 0.2: 86%). Only a small proportion of metabolic features was associated with all three neuropathology scores in the HILIC column (*q* value < 0.2: 2.5%) and C18 column (*q* value < 0.2: 0.49%), indicating potential different metabolic responses across the three measures of AD neuropathology.

**FIGURE 1:**
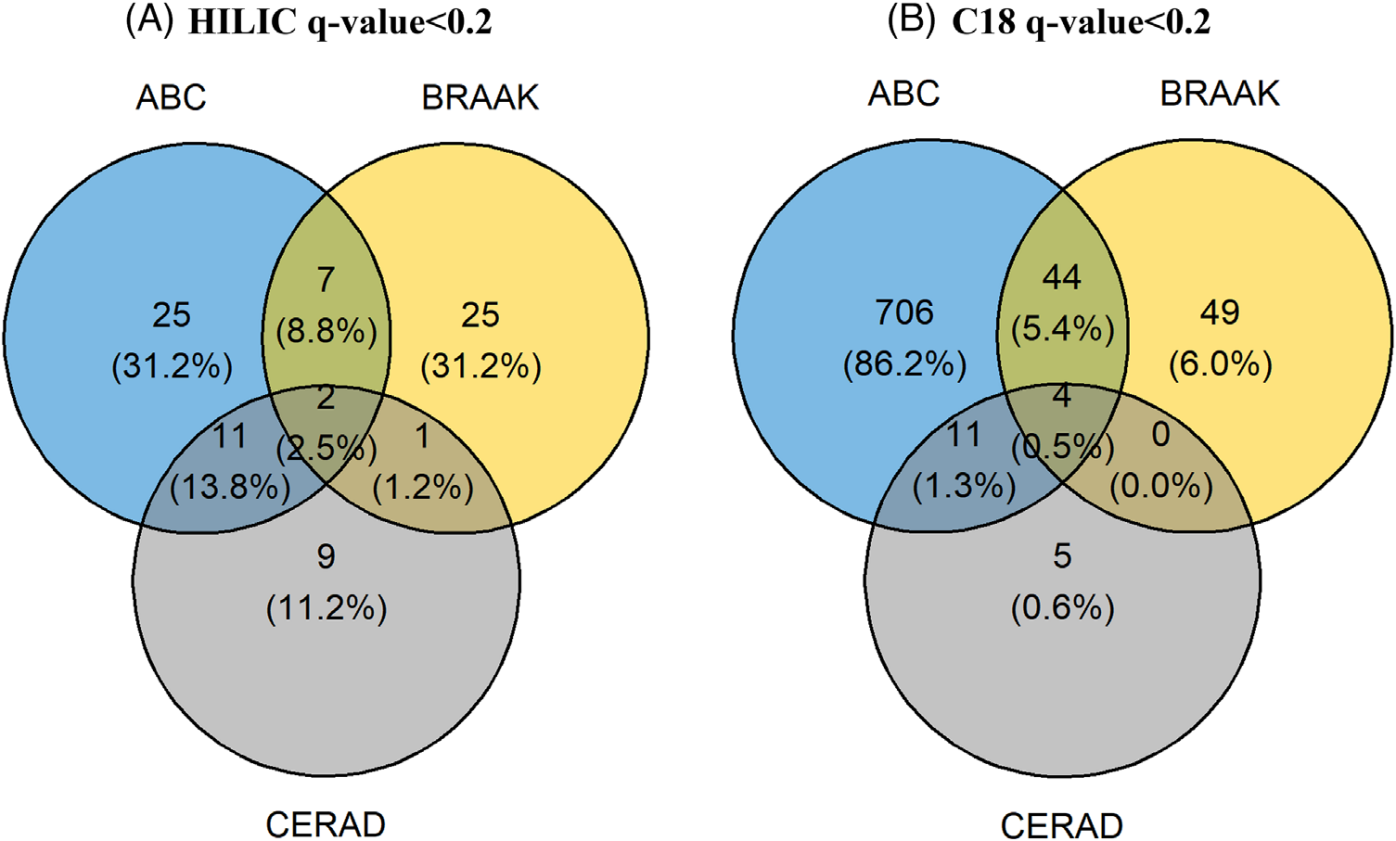
Venn diagram of overlapping metabolic features associated with three Alzheimer's disease neuropathology. Panels A and B present the HILIC and C18 column results with *q*‐values < 0.2. Associations were estimated using an ordinal logistic regression model, adjusted for donors’ race, sex, education, age at death, post‐mortem interval (PMI), and Apoliprotein E genotype. ABC, NIA‐AA Alzheimer's disease neuropathologic change (ADNC); BRAAK, Braak stage for neurofibrillary degeneration; CERAD, Consortium to Establish a Registry for Alzheimer's Disease score for density of neocortical neuritic plaque; HILIC, hydrophilic interaction liquid chromatography.

Metabolic features with raw *p* values < 0.05 were used for pathway enrichment analysis. In total, 36 metabolic pathways were significantly associated with at least one AD neuropathology measure (Figure 1). The enriched pathways pertain to 10 metabolism classes: carbohydrate metabolism, energy metabolism, nucleotide metabolism, amino acid metabolism, lipid metabolism, xenobiotics metabolism, metabolism of cofactors and vitamin, signaling pathway, and digestive system and neurodegenerative disease‐related metabolism.

**FIGURE 2:**
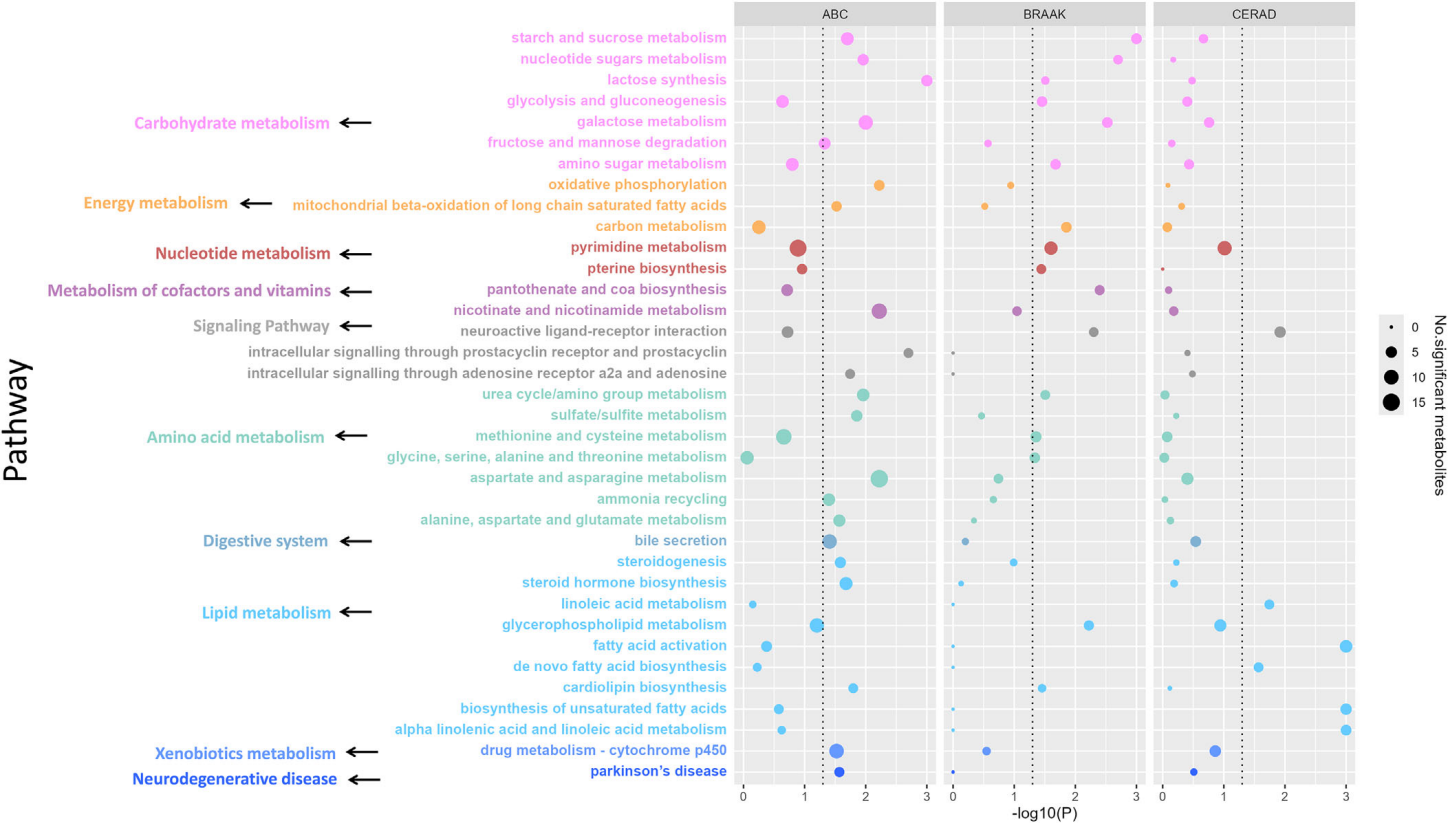
Bubble plots of enrichment pathways associated with three Alzheimer's disease neuropathology. Associations were estimated using an ordinal logistic regression model, adjusted for donors’ race, sex, education, age at death, post‐mortem interval (PMI), and apoliprotein E genotype. Size of bubble represents the number of significant putative metabolites (raw *p* value < 0.05) with m/z matched among each metabolic pathway using *metapone*. The x‐axis represents the negative log10 of *p* value of the association between each significant pathway and outcomes. The color of both bubble and y‐axis label represents the class of each metabolic pathway. The dashed line represents the threshold of *p* value at 0.05. ABC, NIA‐AA Alzheimer's disease neuropathologic change (ADNC); BRAAK, Braak stage for neurofibrillary degeneration; CERAD, Consortium to Establish a Registry for Alzheimer's Disease score for density of neocortical neuritic plaque.

Among the 36 significant pathways, most pathways (*k* = 21) were associated with ABC—a measure of both plaque and tangles, while fewer pathways were perturbed in associations with either Braak (tangles; *k* = 16) or CERAD (plaques; *k* = 6). Nucleotide metabolism, including pyrimidine metabolism and pterin biosynthesis, was exclusively associated with Braak stage. Most amino acid metabolic pathways, such as aspartate and asparagine metabolism, alanine, aspartate and glutamate metabolism, sulfate metabolism, and ammonia recycling were uniquely associated with ABC. Fatty acid‐related pathways, including fatty acid metabolism, de novo fatty acid biosynthesis, biosynthesis of unsaturated fatty acids, and alpha linolenic and linoleic acid metabolism, were only associated with CERAD (Figure 2).

Compared to the authentic compounds analyzed under identical experimental conditions, we confirmed the chemical identities of 18 unique metabolites with Level‐1 evidence, which were nominally significantly associated with at least one neuropathology, with all detected in C18 (Table 3). In sum, about 2% of total significant features were confirmed with level 1 evidence (out of 80 and 819 unique significant features detected in the HILIC and C18 columns, respectively). ABC was significantly associated with all confirmed metabolites, and CERAD with 11 confirmed metabolites. None of the confirmed metabolites were associated with Braak stage. Generally identified metabolites were involved in amino acid metabolism, lipid metabolism, carbohydrate metabolism, nucleotide metabolism, and metabolism of cofactors and vitamins. Specifically, for all metabolites enriched in carbohydrate metabolism, we observed increased intensities of these metabolites positively associated with odds of higher neuropathology markers, with the largest effect being observed in glucose (ABC: odds ratio (OR) [95% confidence interval (CI)] per an interquartile range (IQR) increase in the metabolic feature = 1.59 [1.14, 2.22]; CERAD: OR [95% CI] = 1.74 [1.12, 2.70]). Additionally, several metabolites enriched in purine metabolism, including 1‐methyladenosine, inosine 5′‐phosphate, and guanosine 5′‐diphosphate, were inversely associated with odds of neuropathology change (ABC associated with all three and CERAD associated with guanosine 5′‐diphosphate only), with odds ratios being similar to each other.

**TABLE 3:** Chemical identity^a^ of the metabolic features significantly associated with three AD neuropathologies (raw *p* value < 0.05).

m/z	RT (s)	Metabolite	Pathways	Metabolism	Adduct	Column	AD neuropathology marker	OR (95%CI)^b^
114.0196	14.9	Maleamate	Nicotinate and nicotinamide metabolism	Metabolism of cofactors and vitamins	M‐H	C18	ABC	0.56 (0.32, 0.97)
130.0509	16.1	4‐Hydroxy‐L‐proline	Arginine and proline metabolism	Amino acid metabolism	M‐H	C18	ABC	0.56 (0.33, 0.97)
		N‐Acetyl‐L‐Alanine	Alanine, aspartate, and glutamate metabolism					
140.0117	21	Ethanolamine phosphate	Glycosylphosphatidylinositol‐anchor biosynthesis; Glycerophospholipid metabolism	Lipid metabolism	M‐H	C18	ABC	0.57 (0.34, 0.97)
143.1076	29	Caprylic acid^c^	Fatty acid biosynthesis; Lipoic acid metabolism	Lipid metabolism	M‐H	C18	ABC	2.23 (1.35, 3.68)
174.0408	15.5	N‐Acetyl‐L‐aspartic acid	Alanine, aspartate and glutamate metabolism	Amino acid metabolism	M‐H	C18	ABC	0.54 (0.30, 0.97)
187.1088	20.4	N‐Alpha‐acetyl‐L‐lysine	Lysine biosynthesis	Amino acid metabolism	M‐H	C18	ABC	1.75 (1.12, 2.74)
							CERAD	1.81 (1.08, 3.05)
188.0568	14.4	N‐Acetyl‐L‐glutamic acid^c^	Arginine biosynthesis	Amino acid metabolism	M‐H	C18	ABC	0.45 (0.27, 0.74)
215.033	19.4	Glucose^c^	Glycolysis/Gluconeogenesis; Pentose phosphate pathway; Galactose metabolism; Starch and sucrose metabolism; Amino sugar and nucleotide sugar metabolism	Carbohydrate metabolism	M+Cl	C18	ABC CERAD	1.59 (1.14, 2.22) 1.74 (1.12, 2.70)
259.0229	16.5	D‐Glucose 6‐phosphate^c^	Starch and sucrose metabolism	Carbohydrate metabolism	M‐H	C18	ABC CERAD	1.87 (1.25, 2.79) 1.87 (1.16, 3.00)
		D‐Mannose 6‐phosphate^c^	Fructose and mannose metabolism; Amino sugar and nucleotide sugar metabolism					
		D‐Fructose 6‐phosphate^c^	Galactose metabolism; Starch and sucrose metabolism					
		Alpha‐D‐galactose 1‐phosphate^c^	Galactose metabolism; Amino sugar and nucleotide sugar metabolism					
316.0816	17.3	1‐Methyladenosine^c^	Purine metabolism	Nucleotide metabolism	M+Cl	C18	ABC	0.52 (0.34, 0.80)
347.0401	14.9	Inosine 5′‐phosphate	Purine metabolism	Nucleotide metabolism	M‐H	C18	ABC	0.71 (0.51, 1.00)
442.0176	14.1	Guanosine 5′‐diphosphate^c^	Purine metabolism	Nucleotide metabolism	M‐H	C18	ABC	0.32 (0.18, 0.56)
							CERAD	0.34 (0.17, 0.65)
558.0659	14.6	Adenosine 5′‐Diphosphoribose^c^	Purine metabolism	Nucleotide metabolism	M‐H	C18	ABC	2.01 (1.21, 3.36)
							CERAD	1.92 (1.03, 3.55)
565.0489	14.3	Uridine 5′‐diphosphoglucose	Pentose and glucuronate interconversions; Galactose metabolism; Ascorbate and aldarate metabolism; Starch and sucrose metabolism; Amino sugar and nucleotide sugar metabolism	Carbohydrate metabolism	M‐H	C18	ABC	1.75 (1.24, 2.45)

Abbreviations: ABC, NIA‐AA Alzheimer's disease neuropathologic change (ADNC); AD, Alzheimer's disease; BRAAK, Braak stage for neurofibrillary degeneration; CERAD, Consortium to Establish a Registry for Alzheimer's Disease score for density of neocortical neuritic plaque; CI, confident interval; OR, odds ratio; RT, retention time.

^a^ Chemical identification on the candidate metabolic features was conducted by matching peaks by accurate mass and retention time to authentic reference standards in an in‐house library run under identical conditions using tandem mass spectrometry.

^b^ Odds ratio was estimated using ordinal logistic regression, and model were adjusted for subjects’ race, sex, education, age at death, post‐mortem interval (PMI), and apoliprotein E genotype. The odds ratio was presented per an interquartile range (IQR) increase in the metabolic feature.

^c^ These metabolites were also significant at false discovery rate *q* value < 0.2. Among these, guanosine 5′‐diphosphate was also significant at FDR *q* value < 0.05.

### 3.3 Sensitivity analyses

Several sensitivity analyses were conducted to assess the robustness of our results (Table S2). Three alternative modeling approaches were used in addition to our main OLR model. We used MLR instead of OLR, which considered the neuropathology markers as continuous variables, and additionally adjusted our OLR models for differences in estimated cell type proportions between samples, and technical variation using the first three PCs from the metabolic features. Overall, the number of significant metabolites was similar across all three OLR models, even after including cell type proportions or PCs as additional covariates. The MLR models identified fewer metabolites after adjusting for multiple testing.

Next, we conducted pathway enrichment analyses for the three alternative modeling approaches described above (Figures S1–S3). More enriched pathways (*n* = 50) were significantly associated with at least one AD neuropathology marker when using the MLR in comparison to our main model. Similar to the main analysis, these pathways mainly pertained to 13 metabolism classes, with 10 being the same as in the main analysis and additionally including glycan biosynthesis and metabolism, membrane transport (ATP‐binding cassette [ABC] transporter), and biosynthesis of secondary metabolites. Similar as in the main analysis, ABC was associated with most pathways (*n* = 41) and was exclusively associated with seven amino acid metabolism pathways. Several carbohydrate metabolism pathways, including starch and sucrose metabolism, glycolysis and gluconeogenesis, as well as galactose metabolism, were found to be common pathways among the three neuropathology markers. We also found consistent metabolism classes and pathways when adding the additional covariates of cell type and PCs separately to the OLR models, but with fewer significant metabolites enriched in these pathways and fewer varieties of pathways in each metabolism class. Among these pathways, only ammonia recycling in amino acid metabolism was newly found compared to the main results (Figures S2 and S3).

To evaluate the effect of using different cutoffs of *q*‐ and *p*‐values for the significance in pathway enrichment analysis, we performed sensitivity analyses using *q* value < 0.2 and raw *p* value < 0.01. Similar pathways were enriched when using these different cut‐offs for significance compared to the main analysis (Figure S4).

Finally, we assessed the effect modification of *APOE* genotype for the associations between significant level one metabolites and AD neuropathology markers (Table S3). For most metabolites, the odds ratios were similar regardless of the presence of the *APOE* genotype. The test for interaction only showed significant differences for glucose for both ABC and CERAD (*p* < 0.01) and for adenosine 5′‐diphosphoribose in purine metabolism (CERAD *p* = 0.02), both indicating stronger associations between those metabolites and AD‐related neuropathology markers among participants without an *APOE* ε4 allele.

## 4 DISCUSSION

To our knowledge, this is one of the largest untargeted high‐resolution metabolomics investigations of brain metabolomics perturbations associated with AD neuropathology. We analyzed 162 brain donors, most of whom exhibited advanced AD pathology based on Braak stage, CERAD, and ABC scores. Metabolomic profiling of frontal cortex samples identified 155 significant metabolic features associated with AD markers after adjusting for potentially confounding factors and multiple testing (FDR 5%). Notably, we identified several novel metabolic pathways and confirmed metabolites associated with AD neuropathology, including those related to energy homeostasis (carbohydrate, energy, and lipid metabolism), nucleic acids damage and repair (purine metabolism), and neurotransmission and antioxidant defense (amino acid and neuroactive signaling metabolism).

A key finding from our analyses was the identification of a broad range of metabolic pathways significantly associated with AD neuropathology, shedding light on the complex molecular mechanisms driving AD progression. These pathways spanned across ten distinct metabolic classes, including vital metabolism processes of carbohydrate, energy, nucleotide, amino acid, lipid, xenobiotics, cofactors, and vitamins, signaling, and pathways related to digestion and neurodegenerative disease. Similar pathways have been reported in other metabolomic studies using post‐mortem frontal cortex,^15^ cerebrospinal fluid (CSF),^44^ and plasma samples^45^ from AD patients. The identification of these specific AD‐related metabolic pathways mirror results reported recently. For example, in a multi‐region brain analysis including the cerebellum and temporal cortex, Batra, et al., revealed that methionine cycle, phospholipid metabolism, and glutathione metabolism were commonly regulated in six brain regions and reflected the metabolic dysregulation of AD.^18^ Additional findings from the same study demonstrated alterations in amino acid metabolism pathways, including urea cycle, alanine and aspartate metabolism, tyrosine metabolism, branched‐chain amino acid metabolism, and vitamin metabolism across brain regions.^18^ A recent narrative review further supported that amino acid and lipid metabolism are among the most consistently dysregulated pathways in AD pathogenesis,^8^ underscoring the biological relevance of these findings.

Of particular interest, the Parkinson's disease pathway emerged as significantly associated with the ABC score, indicating potential commonalities in the pathogenic pathways underlying both AD and Parkinson's disease.^46^ Consistent with findings from a previous genome‐wide association study in AD and Parkinson's disease,^47^ our result underscores the complex interplay between neurodegenerative diseases and highlights the importance of investigating shared molecular pathways in understanding their etiology and progression. Furthermore, the neuroactive ligand‐receptor interaction pathway exhibited significant associations with both Braak stage and CERAD, suggesting a potential role of neurotransmitter signaling dysregulation in AD neuropathology.^48^ Dysfunctions in neurotransmitter systems have long been implicated in AD pathogenesis,^49^, ^50^ and our findings provide further evidence supporting the involvement of neuroactive ligand‐receptor interactions in disease progression.

Notably, our analysis revealed differential associations of metabolic features and pathways with each neuropathology measure. ABC score was found to be significantly associated with the largest number of pathways, indicative of its broad representation of AD pathology encompassing amyloid plaque distribution, neuritic plaque, and NFTs.^51^ Braak stage, which reflects the frequency and distribution of NFTs,^29^ showed associations with pathways primarily related to nucleotide metabolism, suggesting potential involvement of these pathways in tau pathology.^52^ Fatty acid‐related pathways were uniquely associated with CERAD, which describes the frequency of neocortical neuritic plaques.^53^

**FIGURE 3:**
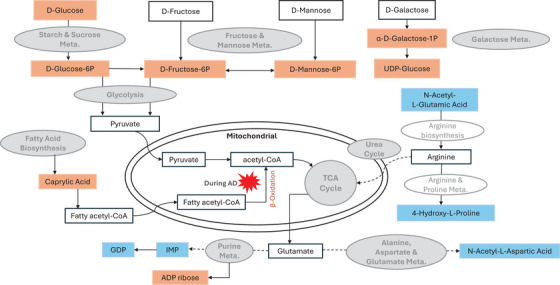
The potential molecular mechanism underlying Alzheimer's disease neuropathology. Metabolites in blue denote a negative association, and metabolites in orange denote a positive association. The gray shaded pathways were found to be significant pathways in enrichment analysis. Other pathways and metabolites are provided for biological context but were not explicitly identified in this research. Solid arrows represent metabolites/pathways directly associated with each other. Dashed lines represent a biochemical pathway linking metabolites not shown for clarity. α‐D‐galactose‐1P, alpha‐D‐galactose 1‐phosphate; ADP ribose, adenosine 5′‐diphosphoribos; D‐fructose‐6P, D‐fructose 6‐phosphate; D‐glucose‐6P, D‐glucose 6‐phosphate; D‐mannose‐6P, D‐mannose 6‐phosphate; GDP, guanosine 5′‐diphosphate; IMP, inosine 5′‐phosphate; Meta., metabolism; UDP‐glucose, uridine 5′‐diphosphoglucose.

The metabolic feature annotation and validation further lent coherence to the pathway analysis. In particular, the confirmation of 18 metabolites with Level 1 evidence provides valuable insights into the specific molecular signatures associated with AD neuropathology (Figure 3). These metabolites, detected exclusively in the C18 hydrophobic column, span a diverse range of metabolic pathways, including key metabolites involved in amino acid metabolism, lipid metabolism, carbohydrate metabolism, nucleotide metabolism, and metabolism of cofactors and vitamins. Prior studies have similarly reported associations between AD phenotypes and metabolites from several chemical classes, including acylcarnitines, amino acids, biogenic amines, and glycerophospholipids.^11^ To further contextualize these findings, we conducted a detailed comparison with previously published AD brain metabolomics studies (Table S4). For example, aspartate and glutamate have been linked to AD onset and progression in the dorsolateral prefrontal cortex.^17^ Our findings build upon this by identifying derivatives of these amino acids, specifically N‐acetyl‐aspartic acid and N‐acetyl‐glutamic acid, which were significantly associated with AD neuropathology. In addition, our results support existing evidence that perturbations in carbohydrate metabolism are central to AD pathology.^54^ Metabolites, including glucose and glucose‐6‐phosphate, have previously been associated with AD‐related metabolic dysfunction, supporting the reproducibility and biological relevance of impaired energy metabolism in AD.^22^ Importantly, our study also provides new insights into purine metabolism, a pathway previously implicated in AD pathophysiology. Prior studies have reported alterations in purine‐related metabolites such as hypoxanthine and uridine.^24^, ^55^ Building on this, we reported novel significant associations between AD neuropathology and these purine‐related metabolites: 1‐methyladenosine, inosine 5′‐phosphate, guanosine 5′‐diphosphate, and adenosine 5′‐diphosphoribose. These findings expand the scope of known purine pathway disruptions in AD neuropathology and highlight potential roles in energy metabolism and nucleotide metabolism, which warrant further functional investigation.

Importantly, besides novel findings in purine metabolism, among 18 level 1 metabolites, other five metabolites were novel and not detected in any of the prior studies, including maleamate, 4‐hydroxy‐L‐proline, N‐acetyl‐L‐alanine, ethanolamine phosphate, N‐alpha‐acetyl‐L‐lysine, uridine, and 5′‐diphosphoglucose. These metabolites, many involved in amino acids and vitamin/cofactor metabolism, were not captured by previous metabolomic studies. The identification of these previously undetected metabolites expands our current understanding of metabolic alterations in AD and suggests additional biological mechanisms that may contribute to the disease progression.

Perturbations in key amino acid metabolism pathways, including aspartate and asparagine metabolism, alanine, aspartate, and glutamate metabolism, sulfate/sulfite metabolism, and ammonia recycling, as well as many related confirmed metabolites, including 4‐hydroxy‐proline, N‐acetyl‐aspartic acid, N‐acetyl‐glutamic acid, and N‐alpha‐acetyl‐lysine, were associated with the ABC score. In general, amino acids serve as essential building blocks for protein synthesis and play crucial roles in various biological processes, including neurotransmission, energy metabolism, and antioxidant defense.^56^ Perturbations in amino acid metabolism have been implicated in neurodegenerative disorders,^57^ including AD,^58^, ^59^, ^60^ due to their involvement in synaptic function, protein aggregation, and oxidative stress.

Among the identified metabolites, those enriched in carbohydrate metabolism exhibited a positive association with the odds of neuropathology change, suggesting a potential role of glucose dysregulation in AD pathogenesis (Figure 3).^61^ Specifically, elevated glucose levels were linked to greater neuropathology, consistent with previous studies.^54^, ^62^ When brain glycolysis is impaired, fatty acid oxidation may compensate by producing acetyl‐CoA in the TCA cycle to maintain energy homeostasis.^54^, ^63^ Metabolomics research has also shown altered energy homeostasis, including TCA cycle, lipid metabolism, and mitochondrial ketone bodies, in plasma and CSF samples.^64^, ^65^ Thus, elevated carbohydrate metabolites in our study may reflect decreased glucose utilization and a shift toward alternative energy sources, including fatty acids. Interestingly, this association was particularly strong among *APOE* ε4 allele non‐carriers.

We also found metabolites enriched in purine metabolism—a pathway involved in DNA damage repair, including 1‐methyladenosine, inosine 5′‐phosphate, and guanosine 5′‐diphosphate, to be negatively associated with neuropathology. This suggests purine metabolism may help mitigate AD pathology,^66^ possibly via neuroprotection and synaptic function regulation.^67^ Again, this pattern was stronger in *APOE* ε4 allele non‐carriers.

The strength of our study lies in the comprehensive approach we employed, integrating various methodologies and analytical techniques to elucidate the metabolic perturbations associated with AD neuropathology. First, we utilized an untargeted high‐resolution metabolomics platform coupled with comprehensive metabolomics profiling. Specifically, our current platform detected over 36,000 metabolic features across both HILIC and C18 columns, offering greater analytical depth than other untargeted platforms, which typically report fewer than 1000 features and rely on predefined compound libraries and narrower chromatographic windows. The biological interpretation of these 36,000 metabolic features was facilitated using two complementary approaches: (1) We used a novel bioinformatic platform to predict functional biological activities of the metabolites, without relying on identified or validated metabolites for pathway mapping^41^ and (2) we confirmed around 600 metabolites with authentic standards, enabling high‐confidence (Level 1) identification.^43^ This methodological combination enabled us to identify and characterize metabolic features and pathways associated with AD neuropathology markers, providing insights into the molecular mechanisms underlying AD pathogenesis. Furthermore, our study extends beyond traditional analyses by investigating metabolic alterations directly in brain tissue samples, a significant advancement over studies relying solely on peripheral biomarkers. Third, our research protocol included three types of robust phenotypic measures of AD neuropathology, minimizing the potential for misclassification bias.

Although our findings are biologically plausible and statistically robust, several common limitations for metabolomics studies of AD neuropathology should be acknowledged. First, the cross‐sectional design precludes the establishment of causality and the directionality of observed associations between metabolic features and AD markers. While longitudinal studies are not feasible with post‐mortem brain tissue, characterizing metabolic changes before death (e.g., in CSF) could offer insights into the temporal dynamics of AD‐related alterations. Future work should also explore how brain metabolomic signatures relate to *pre mortem* cognitive evaluations. Second, brain metabolism is inherently dynamic and may be influenced by post‐mortem factors or unmeasured biological or environmental variables. While we employed stringent quality control and statistical adjustments, residual or unmeasured confounding cannot be ruled out. Factors such as comorbidities, obesity, nutrition, medication use, and lifestyle factors may impact both metabolic profiles and AD neuropathology. Third, metabolite annotation was based on an existing serum metabolites database, which may not comprehensively capture the unique metabolic landscape of prefrontal cortex tissue. Limitations in analytical standard libraries also constrain identification accuracy. More comprehensive metabolomic databases and pathway annotations will be essential for gaining deeper molecular insights. Additionally, our analysis focused solely on prefrontal cortex samples. While this region is relevant to AD, other brain areas (e.g., the hippocampus) could provide a more comprehensive understanding of the brain‐wide metabolomic changes. Although our sample size was sufficient for detecting significant associations, replication in an independent brain bank is warranted. Our cohort was predominantly well‐educated and White, potentially limiting generalizability. Future studies with larger, more diverse populations are needed. Last, the Emory ADRC brain bank is a convenience sample enriched for AD and related conditions, not a population‐based cohort, which may influence representativeness.

## 5 CONCLUSION

In this comprehensive brain metabolomics investigation on AD neuropathology markers, we identified numerous metabolic features and pathways significantly associated with AD neuropathology. We also confirmed several Level‐1 metabolites involved in amino acid, lipid, carbohydrate, nucleotide, and cofactors and vitamins metabolism. Notably, some confirmed metabolites showed stronger associations among *APOE* ε4 non‐carriers, who had greater variability in neuropathology markers than *APOE* e4 carriers. These findings enhance our understanding of AD‐related metabolic alterations and highlight the potential of metabolomic profiling to reveal disease mechanisms. Further studies are needed to validate and explore the functional roles of these metabolites in AD progression.

## CONFLICT OF INTEREST STATEMENT

The authors declare no conflicts of interest. Author disclosures are available in the supporting information.

## CONSENT STATEMENT

All relevant ethical guidelines have been followed, and any necessary institutional review board and/or ethics committee approvals have been obtained. Written informed consent was obtained from all participants before inclusion in the study.

## Supporting information



Supporting Information

Supporting Information

Supporting Information

## References

[1] 2021 Alzheimer's disease facts and figures. Alzheimers Dement. 2021;17:327‐406. 33756057 10.1002/alz.12328

[2] Xu J , Murphy SL , Kochanek KD , Arias E . Mortality in the United States, 2018 Key Findings Data from the National Vital Statistics System. Accessed July 24, 2024. https://www.cdc.gov/nchs/data/databriefs/db355-h.pdf

[3] 2022 Alzheimer's disease facts and figures. Alzheimers Dement. 2022;18:700‐789. 35289055 10.1002/alz.12638

[4] Zhang J . Recent advances in Alzheimer's disease: mechanisms, clinical trials and new drug development strategies. Signal Transduct Target Ther. 2024;9:211. 39174535 10.1038/s41392-024-01911-3 PMC11344989

[5] Zhang Y , Chen H , Li R , Sterling K , Song W . Amyloid β‐based therapy for Alzheimer's disease: challenges, successes and future. Signal Transduct Target Ther. 2023;8:248. 37386015 10.1038/s41392-023-01484-7 PMC10310781

[6] Dhapola R , Beura SK , Sharma P , Singh SK , HariKrishnaReddy D . Oxidative stress in Alzheimer's disease: current knowledge of signaling pathways and therapeutics. Mol Biol Rep. 2024;51:48. 38165499 10.1007/s11033-023-09021-z

[7] Clarke JR , Ribeiro FC , Frozza RL , De Felice FG , Lourenco MV . Metabolic dysfunction in Alzheimer's disease: from basic neurobiology to clinical approaches. J Alzheimers Dis. 2018;64:S405‐S426. 29562518 10.3233/JAD-179911

[8] Lista S , González‐Domínguez R , López‐Ortiz S , et al. Integrative metabolomics science in Alzheimer's disease: relevance and future perspectives. Ageing Res Rev. 2023;89:101987. 37343679 10.1016/j.arr.2023.101987 PMC12450062

[9] Amidfar M , Askari G , Kim Y‐K . Association of metabolic dysfunction with cognitive decline and Alzheimer's disease: a review of metabolomic evidence. Prog Neuropsychopharmacol Biol Psychiatry. 2024;128:110848. 37634657 10.1016/j.pnpbp.2023.110848

[10] Varma VR , Oommen AM , Varma S , et al. Brain and blood metabolite signatures of pathology and progression in Alzheimer disease: a targeted metabolomics study. PLoS Med. 2018;15:e1002482. 29370177 10.1371/journal.pmed.1002482 PMC5784884

[11] Huo Z , Yu L , Yang J , et al. Brain and blood metabolome for Alzheimer's dementia: findings from a targeted metabolomics analysis. Neurobiol Aging. 2020;86:123‐133. 31785839 10.1016/j.neurobiolaging.2019.10.014 PMC6995427

[12] Mahajan UV , Varma VR , Griswold ME , et al. Dysregulation of multiple metabolic networks related to brain transmethylation and polyamine pathways in Alzheimer disease: a targeted metabolomic and transcriptomic study. PLoS Med. 2020;17:e1003439. 31978055 10.1371/journal.pmed.1003012 PMC6980402

[13] Kalecký K , German DC , Montillo AA , Bottiglieri T . Targeted metabolomic analysis in Alzheimer's disease plasma and brain tissue in non‐Hispanic whites. J Alzheimers Dis. 2022;86:1875‐1895. 35253754 10.3233/JAD-215448 PMC9108583

[14] Ansoleaga B , Jové M , Schlüter A , et al. Deregulation of purine metabolism in Alzheimer's disease. Neurobiol Aging. 2015;36:68‐80. 25311278 10.1016/j.neurobiolaging.2014.08.004

[15] Paglia G , Stocchero M , Cacciatore S , et al. Unbiased metabolomic investigation of Alzheimer's disease brain points to dysregulation of mitochondrial aspartate metabolism. J Proteome Res. 2016;15:608‐618. 26717242 10.1021/acs.jproteome.5b01020 PMC5751881

[16] Snowden SG , Ebshiana AA , Hye A , et al. Association between fatty acid metabolism in the brain and Alzheimer disease neuropathology and cognitive performance: a nontargeted metabolomic study. PLoS Med. 2017;14:e1002266. 28323825 10.1371/journal.pmed.1002266 PMC5360226

[17] Novotny BC , Fernandez MV , Wang C , et al. Metabolomic and lipidomic signatures in autosomal dominant and late‐onset Alzheimer's disease brains. Alzheimers Dement. 2023;19:1785‐1799. 36251323 10.1002/alz.12800 PMC10106526

[18] Batra R , Krumsiek J , Wang X , et al. Brain region‐specific metabolic signatures of Alzheimer's disease. Alzheimers Dement. 2022;18:e067879.

[19] Batra R , Krumsiek J , Wang X , et al. Comparative brain metabolomics reveals shared and distinct metabolic alterations in Alzheimer's disease and progressive supranuclear palsy. Alzheimers Dement. 2024;20:8294‐8307. 39439201 10.1002/alz.14249 PMC11667510

[20] Batra R , Arnold M , Wörheide MA , et al. The landscape of metabolic brain alterations in Alzheimer's disease. Alzheimers Dement. 2023;19:980‐998. 35829654 10.1002/alz.12714 PMC9837312

[21] Shao Y , Ouyang Y , Li T , et al. Alteration of metabolic profile and potential biomarkers in the plasma of Alzheimer's disease. Aging Dis. 2020;11:1459‐1470. 33269100 10.14336/AD.2020.0217 PMC7673846

[22] Eteleeb AM , Novotny BC , Tarraga CS , et al. Brain high‐throughput multi‐omics data reveal molecular heterogeneity in Alzheimer's disease. PLoS Biol. 2024;22:e3002607. 38687811 10.1371/journal.pbio.3002607 PMC11086901

[23] Panyard DJ , Kurti SP , Stienmetz BM , et al. Cerebrospinal fluid metabolomics identifies 19 brain‐related phenotype associations. Commun Biol. 2021;4:63. 33437055 10.1038/s42003-020-01583-z PMC7803963

[24] François M , Karpe AV , Liu JW , et al. Multi‐omics, an integrated approach to identify novel blood biomarkers of Alzheimer's disease. Metabolites. 2022;12:949. 36295851 10.3390/metabo12100949 PMC9610280

[25] Christensen GM , Li Z , Liang D , et al. Association of PM2.5 exposure and Alzheimer disease pathology in brain bank donors‐effect modification by APOE genotype. Neurology. 2024;102:e209162. 38382009 10.1212/WNL.0000000000209162 PMC13084541

[26] Li Z , Liang D , Ebelt S , et al. Differential DNA methylation in the brain as potential mediator of the association between traffic‐related PM2.5 and neuropathology markers of Alzheimer's disease. Alzheimers Dement. 2024;20:2538‐2551. 38345197 10.1002/alz.13650 PMC11032571

[27] Pett L , Li Z , Abrishamcar S , et al. The association between neighborhood deprivation and DNA methylation in an autopsy cohort. Aging. 2024;16(8):6694‐6716. doi:10.18632/aging.205764 38663907 PMC11087100

[28] Besser LM , Kukull WA , Teylan MA , et al. The revised National Alzheimer's Coordinating Center's neuropathology form‐available data and new analyses. J Neuropathol Exp Neurol. 2018;77:717‐726. 29945202 10.1093/jnen/nly049 PMC6044344

[29] Braak H , Braak E . Neuropathological stageing of Alzheimer‐related changes. Acta Neuropathol. 1991;82:239‐259. 1759558 10.1007/BF00308809

[30] Mirra SS , Heyman A , McKeel D , et al. The consortium to establish a registry for Alzheimer's disease (CERAD). Part II. Standardization of the neuropathologic assessment of Alzheimer's disease. Neurology. 1991;41:479‐486. 2011243 10.1212/wnl.41.4.479

[31] Montine TJ , Phelps CH , Beach TG , et al. National Institute on Aging–Alzheimer's Association guidelines for the neuropathologic assessment of Alzheimer's disease: a practical approach. Acta Neuropathol. 2012;123:1‐11. 22101365 10.1007/s00401-011-0910-3 PMC3268003

[32] Thal DR , Rüb U , Orantes M , Braak H . Phases of Aβ‐deposition in the human brain and its relevance for the development of AD. Neurology. 2002;58:1791‐1800. 12084879 10.1212/wnl.58.12.1791

[33] Go YM , Walker DI , Liang Y , et al. Reference standardization for mass spectrometry and high‐resolution metabolomics applications to exposome research. Toxicol Sci. 2015;148:531‐543. 26358001 10.1093/toxsci/kfv198 PMC4675836

[34] Ladva CN , Golan R , Liang D , et al. Particulate metal exposures induce plasma metabolome changes in a commuter panel study. PLoS One. 2018;13:1‐19. 10.1371/journal.pone.0203468 PMC6145583 30231074

[35] Liang D , Ladva CN , Golan R , et al. Perturbations of the arginine metabolome following exposures to traffic‐related air pollution in a panel of commuters with and without asthma. Environ Int. 2019;127:503‐513. 30981021 10.1016/j.envint.2019.04.003 PMC6513706

[36] Liang D , Moutinho JL , Golan R , et al. Use of high‐resolution metabolomics for the identification of metabolic signals associated with traffic‐related air pollution. Environ Int. 2018;120:145‐154. 30092452 10.1016/j.envint.2018.07.044 PMC6414207

[37] Ribbenstedt A , Ziarrusta H , Benskin JP . Development, characterization and comparisons of targeted and non‐targeted metabolomics methods. PLoS One. 2018;13:e0207082. 30439966 10.1371/journal.pone.0207082 PMC6237353

[38] Uppal K , Soltow QA , Strobel FH , et al. xMSanalyzer: automated pipeline for improved feature detection and downstream analysis of large‐scale, non‐targeted metabolomics data. BMC Bioinformatics. 2013;14(1):15. 23323971 10.1186/1471-2105-14-15 PMC3562220

[39] Yu T , Park Y , Johnson JM , Jones DP . apLCMS—adaptive processing of high‐resolution LC/MS data. Bioinformatics. 2009;25:1930‐1936. 19414529 10.1093/bioinformatics/btp291 PMC2712336

[40] Ripley B , Venables W . nnet: feed‐forward neural networks and multinomial log‐linear models. R package version 7.3‐12.2017.

[41] Tian L , Li Z , Ma G , et al. Metapone: a Bioconductor package for joint pathway testing for untargeted metabolomics data. Bioinformatics. 2022;38:3662‐3664. 35639952 10.1093/bioinformatics/btac364 PMC9272804

[42] Morrison N , Bearden D , Bundy JG , et al. Standard reporting requirements for biological samples in metabolomics experiments: environmental context. Metabolomics. 2007;3:203‐210.

[43] Liu KH , Nellis M , Uppal K , et al. Reference standardization for quantification and harmonization of large‐scale metabolomics. Anal Chem. 2020;92:8836‐8844. 32490663 10.1021/acs.analchem.0c00338 PMC7887762

[44] Dong R , Denier‐Fields DN , Lu Q , et al. Principal components from untargeted cerebrospinal fluid metabolomics associated with Alzheimer's disease biomarkers. Neurobiol Aging. 2022;117:12‐23. 35640460 10.1016/j.neurobiolaging.2022.04.009 PMC9737218

[45] Milos T , Rojo D , Nedic Erjavec G , et al. Metabolic profiling of Alzheimer's disease: untargeted metabolomics analysis of plasma samples. Prog Neuropsychopharmacol Biol Psychiatry. 2023;127:110830. 37454721 10.1016/j.pnpbp.2023.110830

[46] Tan SH , Karri V , Tay NWR , et al. Emerging pathways to neurodegeneration: dissecting the critical molecular mechanisms in Alzheimer's disease, Parkinson's disease. Biomed Pharmacother. 2019;111:765‐777. 30612001 10.1016/j.biopha.2018.12.101

[47] Ramanan VK , Saykin AJ . Pathways to neurodegeneration: mechanistic insights from GWAS in Alzheimer's disease, Parkinson's disease, and related disorders. Am J Neurodegener Dis. 2013;2:145‐175. 24093081 PMC3783830

[48] Abyadeh M , Tofigh N , Hosseinian S , et al. Key genes and biochemical networks in various brain regions affected in Alzheimer's disease. Cells. 2022;11(6):987. 35326437 10.3390/cells11060987 PMC8946735

[49] Xu Y , Yan J , Zhou P , et al. Neurotransmitter receptors and cognitive dysfunction in Alzheimer's disease and Parkinson's disease. Prog Neurobiol. 2012;97:1‐13. 22387368 10.1016/j.pneurobio.2012.02.002 PMC3371373

[50] Paula‐Lima AC , Brito‐Moreira J , Ferreira ST . Deregulation of excitatory neurotransmission underlying synapse failure in Alzheimer's disease. J Neurochem. 2013;126:191‐202. 23668663 10.1111/jnc.12304

[51] Corey‐Bloom J . The ABC of Alzheimer's disease: cognitive changes and their management in Alzheimer's disease and related dementias. Int Psychogeriatr. 2002;14(1):51‐75. 12636180 10.1017/s1041610203008664

[52] Iqbal K , del Alonso CA , Chen S , et al. Tau pathology in Alzheimer disease and other tauopathies. Biochim Biophys Acta. 2005;1739:198‐210. 15615638 10.1016/j.bbadis.2004.09.008

[53] Fillenbaum GG , van Belle G , Morris JC , et al. Consortium to establish a registry for Alzheimer's disease (CERAD): the first twenty years. Alzheimers Dement. 2008;4:96‐109. 18631955 10.1016/j.jalz.2007.08.005 PMC2808763

[54] Wilkins JM , Trushina E . Application of metabolomics in Alzheimer's disease. Front Neurol. 2017;8:719. 29375465 10.3389/fneur.2017.00719 PMC5770363

[55] Hajjar I , Liu C , Jones DP , Uppal K . Untargeted metabolomics reveal dysregulations in sugar, methionine, and tyrosine pathways in the prodromal state of AD. Alzheimers Dement (Amst). 2020;12:e12064. 32793799 10.1002/dad2.12064 PMC7418891

[56] Fagg GE , Foster AC . Amino acid neurotransmitters and their pathways in the mammalian central nervous system. Neuroscience. 1983;9:701‐719. 6137788 10.1016/0306-4522(83)90263-4

[57] Kori M , Aydın B , Unal S , Arga KY , Kazan D . Metabolic biomarkers and neurodegeneration: a pathway enrichment analysis of Alzheimer's Disease, Parkinson's Disease, and amyotrophic lateral sclerosis. OMICS. 2016;20:645‐661. 27828769 10.1089/omi.2016.0106

[58] van der Velpen V , Teav T , Gallart‐Ayala H , et al. Systemic and central nervous system metabolic alterations in Alzheimer's disease. Alzheimers Res Ther. 2019;11:93. 31779690 10.1186/s13195-019-0551-7 PMC6883620

[59] Xu J , Begley P , Church SJ , et al. Graded perturbations of metabolism in multiple regions of human brain in Alzheimer's disease: snapshot of a pervasive metabolic disorder. Biochim Biophys Acta. 2016;1862:1084‐1092. 26957286 10.1016/j.bbadis.2016.03.001 PMC4856736

[60] González‐Domínguez R , García‐Barrera T , Gómez‐Ariza JL . Metabolite profiling for the identification of altered metabolic pathways in Alzheimer's disease. J Pharm Biomed Anal. 2015;107:75‐81. 25575172 10.1016/j.jpba.2014.10.010

[61] An Y , Varma VR , Varma S , et al. Evidence for brain glucose dysregulation in Alzheimer's disease. Alzheimers Dement. 2018;14:318‐329. 29055815 10.1016/j.jalz.2017.09.011 PMC5866736

[62] Poddar MK , Banerjee S , Chakraborty A , Dutta D . Metabolic disorder in Alzheimer's disease. Metab Brain Dis. 2021;36:781‐813. 33638805 10.1007/s11011-021-00673-z

[63] Heininger K . A unifying hypothesis of Alzheimer's disease. IV. Causation and sequence of events. Rev Neurosci. 2000;11 Spec No:213‐328. 11065271 10.1515/revneuro.2000.11.s1.213

[64] Kaddurah‐Daouk R , Zhu H , Sharma S , et al. Alterations in metabolic pathways and networks in Alzheimer's disease. Transl Psychiatry. 2013;3:e244. 23571809 10.1038/tp.2013.18 PMC3641405

[65] Han X , Rozen S , Boyle SH , et al. Metabolomics in early Alzheimer's disease: identification of altered plasma sphingolipidome using shotgun lipidomics. PLoS One. 2011;6:e21643. 21779331 10.1371/journal.pone.0021643 PMC3136924

[66] Williams HC , Farmer BC , Piron MA , et al. APOE alters glucose flux through central carbon pathways in astrocytes. Neurobiol Dis. 2020;136:104742. 31931141 10.1016/j.nbd.2020.104742 PMC7044721

[67] Tang Z , Ye W , Chen H , et al. Role of purines in regulation of metabolic reprogramming. Purinergic Signal. 2019;15:423‐438. 31493132 10.1007/s11302-019-09676-z PMC6923298

