# Supplementary material for: Metabolic dysregulation in Alzheimer's disease: A brain metabolomics approach
Source: Alzheimers Dement. 2025 Sep 11;21(9):e70528. doi: 10.1002/alz.70528 (PMC12423944; doi:10.1002/alz.70528)
Supplement: Supplementary file 2 — Supporting Information [file ALZ-21-e70528-s003.docx]

**Supplementary Materials**

**Metabolic Dysregulation in Alzheimer's Disease: A Brain Metabolomics Approach**

Anke Hüls^1,2,3,4^ **^†^**_,_ Youran Tan^2^ **^†^**, Emma Casey^1^, Zhenjiang Li^2^, Marla Gearing^4,5,6^, Allan I. Levey^4,6^, James J. Lah^4,5^, Aliza P. Wingo^7,8^, Dean P Jones^9^, Douglas I Walker^2^, Thomas S. Wingo^10,11^, Donghai Liang^1,2^

**^†^These authors contributed equally to this work.**

**Author affiliations:**

^1^ Department of Epidemiology, Rollins School of Public Health, Emory University, Atlanta, Georgia 30322, USA

^2^ Gangarosa Department of Environmental Health, Rollins School of Public Health, Emory University, Atlanta, Georgia 30322, USA

^3^ Department of Biostatistics and Bioinformatics, Rollins School of Public Health, Emory University, Atlanta, Georgia 30322, USA

^4^ Goizueta Alzheimer's Disease Research Center, School of Medicine, Emory University, Atlanta, GA 30322, USA

^5^ Department of Pathology and Laboratory Medicine, Emory University, Atlanta, Georgia 30322, USA

^6^ Department of Neurology, Emory University School of Medicine, Atlanta, Georgia 30322, USA

^7^ Department of Psychiatry, University of California, Davis, Sacramento, CA USA

^8^ Division of Mental Health, Northern California VA, Sacramento, CA 95816, USA

^9^ Department of Medicine, Emory University, Atlanta, Georgia 30322, USA

^10^ Department of Neurology, University of California, Davis, Sacramento, CA 95816, USA

^11^ Alzheimer’s Disease Research Center, University of California, Davis, Sacramento, CA 95816, USA

**Correspondence to:**

Anke Huels, PhD

Full address: Department of Epidemiology, Rollins School of Public Health, Emory University, 1518 Clifton Rd NE, Atlanta, GA, 30322, USA

E-mail: [anke.huels@emory.edu](mailto:anke.huels@emory.edu)

Donghai Liang, PhD

Full address: Gangarosa Department of Environmental Health, Rollins School of Public Health, Emory University, 1518 Clifton Rd NE, Atlanta, GA, 30322, USA

E-mail: [donghai.liang@emory.edu](mailto:donghai.liang@emory.edu)

**Running title**: Metabolomic perturbation of Alzheimer's disease

**Keywords:** Alzheimer’s disease; neuropathology; untargeted metabolomics; high-resolution metabolomics

**Table S2.** Number of metabolic features associated with three Alzheimer's disease neuropathology in sensitivity analyses.

| Model type | Outcome | HILIC ESI+ (No. metabolic features =20051) | | | | C18 ESI- (No. metabolic features =15927) | | | |
| --- | --- | --- | --- | --- | --- | --- | --- | --- | --- |
|  |  | FDR  q-value<0.05 | FDR  q-value<0.2 | Raw  p-value<0.01 | Raw  p-value<0.05 | FDR  q-value<0.05 | FDR  q-value<0.2 | Raw  p-value<0.01 | Raw  p-value<0.05 |
| OLR (main model) | ABC | 26 | 45 | 457 | 1712 | 64 | 765 | 756 | 2115 |
|  | Braak stage | 17 | 35 | 174 | 606 | 12 | 97 | 92 | 305 |
|  | CERAD | 20 | 23 | 191 | 1001 | 16 | 20 | 193 | 890 |
| MLR | ABC | 0 | 0 | 389 | 1597 | 0 | 206 | 580 | 1931 |
|  | Braak stage | 0 | 0 | 435 | 1698 | 4 | 392 | 654 | 1956 |
|  | CERAD | 0 | 1 | 274 | 1326 | 0 | 0 | 272 | 1232 |
| OLR  cell type | ABC | 20 | 35 | 392 | 1553 | 66 | 803 | 755 | 2117 |
|  | Braak stage | 14 | 26 | 510 | 2007 | 18 | 209 | 213 | 599 |
|  | CERAD | 22 | 24 | 210 | 1034 | 17 | 25 | 236 | 1022 |
| OLR  PC | ABC | 35 | 66 | 419 | 1612 | 89 | 997 | 836 | 2254 |
|  | Braak stage | 25 | 63 | 173 | 564 | 27 | 136 | 112 | 321 |
|  | CERAD | 20 | 21 | 87 | 442 | 18 | 19 | 72 | 301 |

Abbreviations: ABC: NIA-AA Alzheimer's disease neuropathologic change (ADNC); BRAAK: Braak stage for neurofibrillary degeneration; CERAD: CERAD score for density of neocortical neuritic plaque; MLR: multivariable linear regression; OLR: ordinal logistic regression; PC: principal component.

Note: FDR indicates Benjamini-Hochberg procedure for false discovery rate correction of multiple comparisons.

Models were adjusted for subjects’ race, sex, education, age at death, postmortem interval and apoliprotein-E genotype.

OLR (main model): Results from the main analysis (compare Table 2).

MLR: MLR was used instead of OLR, which considered the neuropathology markers as continuous variables.

OLR cell type: OLR additionally adjusted for estimated cell type proportions.

OLR PC: OLR additionally adjusted for the first three principal components from the metabolic features to control for potential batch effects.

**Table S3**. Effect modification of APOE on the metabolic features significantly associated with three Alzheimer's disease neuropathology (raw p-value <0.05).

| **m/z** | **RT (s)** | **Metabolite** | **OR (95%CI)** | | | **P for interaction** |
| --- | --- | --- | --- | --- | --- | --- |
|  |  |  | **Main analysis^#^** | **APOE (absence of E4)^^^** | **APOE (presence of E4)^^^** |  |
| 114.0196 | 14.9 | Maleamate | ABC: 0.56 (0.32, 0.97) | ABC: 0.54 (0.35, 0.85) | ABC: 0.57 (0.29, 1.11) | ABC: 0.71 |
| 130.0509 | 16.1 | 4-Hydroxy-L-Proline | ABC: 0.56 (0.33, 0.97) | ABC: 0.54 (0.35, 0.83) | ABC: 0.58 (0.30, 1.12) | ABC: 0.53 |
|  |  | N-Acetyl-L-Alanine |  |  |  |  |
| 140.0117 | 21 | Ethanolamine Phosphate | ABC: 0.57 (0.34, 0.97) | ABC: 0.59 (0.40, 0.87) | ABC: 0.54 (0.29, 1.01) | ABC: 0.49 |
| 143.1076 | 29 | Caprylic Acid^*^ | ABC: 2.23 (1.35, 3.68) | ABC: 1.93 (1.09, 3.41) | ABC: 3.03 (1.21, 7.57) | ABC: 0.40 |
| 174.0408 | 15.5 | N-Acetyl-L-Aspartic Acid | ABC: 0.54 (0.30, 0.97) | ABC: 0.52 (0.33, 0.81) | ABC: 0.57 (0.28, 1.14) | ABC: 0.46 |
| 187.1088 | 20.4 | N-Alpha-Acetyl-L-Lysine | ABC: 1.75 (1.12, 2.74) | ABC: 2.27 (1.24, 4.14) | ABC: 1.29 (0.66, 2.50) | ABC: 0.22 |
|  |  |  | CERAD: 1.81 (1.08, 3.05) | CERAD: 2.39 (1.23, 4.66) | CERAD: 1.06 (0.43, 2.61) | CERAD: 0.16 |
| 188.0568 | 14.4 | N-Acetyl-L-Glutamic Acid^*^ | ABC: 0.45 (0.27, 0.74) | ABC: 0.42 (0.29, 0.61) | ABC: 0.47 (0.26, 0.85) | ABC: 0.33 |
| 215.033 | 19.4 | Glucose^*^ | ABC: 1.59 (1.14, 2.22)  CERAD: 1.74 (1.12, 2.70) | ABC: 2.05 (1.59, 2.63)  CERAD: 3.81 (2.56, 5.69) | ABC: 1.24 (0.80, 1.92)  CERAD: 0.68 (0.35, 1.33) | ABC: <0.01 CERAD: <0.01 |
| 259.0229 | 16.5 | D-Glucose 6-Phosphate^*^ | ABC: 1.87 (1.25, 2.79)  CERAD: 1.87 (1.16, 3.00) | ABC: 2.21 (1.22, 4.00)  CERAD: 2.45 (1.28, 4.69) | ABC: 1.65 (0.95, 2.84)  CERAD: 1.24 (0.56, 2.78) | ABC: 0.47  CERAD: 0.20 |
|  |  | D-Mannose 6-Phosphate^*^ |  |  |  |  |
|  |  | D-Fructose 6-Phosphate^*^ |  |  |  |  |
|  |  | Alpha-D-Galactose 1-Phosphate^*^ |  |  |  |  |
| 316.0816 | 17.3 | 1-Methyladenosine^*^ | ABC: 0.52 (0.34, 0.80) | ABC: 0.53 (0.31, 0.90) | ABC: 0.50 (0.25, 0.99) | ABC: 0.88 |
| 347.0401 | 14.9 | Inosine 5'-Phosphate | ABC: 0.71 (0.51, 1.00) | ABC: 0.66 (0.43, 1.03) | ABC: 0.77 (0.47, 1.27) | ABC: 0.64 |
| 442.0176 | 14.1 | Guanosine 5'-Diphosphate^*^ | ABC: 0.32 (0.18, 0.56) | ABC: 0.33 (0.17, 0.63) | ABC: 0.32 (0.13, 0.79) | ABC: 0.95 |
|  |  |  | CERAD: 0.34 (0.17, 0.65) | CERAD: 0.33 (0.16, 0.68) | CERAD: 0.40 (0.13, 1.23) | CERAD: 0.76 |
| 558.0659 | 14.6 | Adenosine 5'-Diphosphoribose^*^ | ABC: 2.01 (1.21, 3.36) | ABC: 2.86 (1.34, 6.11) | ABC: 1.46 (0.72, 2.94) | ABC: 0.20 |
|  |  |  | CERAD: 1.92 (1.03, 3.55) | CERAD: 2.68 (1.46, 4.92) | CERAD: 0.89 (0.45, 1.78) | CERAD: 0.02 |
| 565.0489 | 14.3 | Uridine 5'-Diphosphoglucose | ABC: 1.75 (1.24, 2.45) | ABC: 2.20 (1.38, 3.52) | ABC: 1.34 (0.81, 2.22) | ABC: 0.15 |

Abbreviations: APOE: apolipoprotein E; ABC: NIA-AA Alzheimer's disease neuropathologic change (ADNC); BRAAK: Braak stage for neurofibrillary degeneration; CERAD: CERAD score for density of neocortical neuritic plaque; OR: odds ratio; CI: confident interval;

**^#^** Odds ratio was estimated using ordinal logistic regression, and model were adjusted for subjects’ race, sex, education, age at death, postmortem interval and apoliprotein-E genotype; The odds ratio was presented per an interquartile range (IQR) increase in the metabolic feature.

^ Odds ratio was estimated using ordinal logistic regression, with multiplicative interaction term between the significant level one metabolic and APOE genotype (presence or absence of ε4 allele); The odds ratio was presented per an interquartile range (IQR) increase in the metabolic feature.

**^*^** These metabolites were also significant at FDR q-value<0.2. Among these, Guanosine 5'-Diphosphate was also significant at FDR q-value<0.05.

**Table S4.** The summary of prior AD related metabolomics studies on human published after 2018.

| **Title** | **Outcome assessment** | **Approach** | **Study population** | **Study sample** | **Range of metabolites detected** | **Main results (common pathways; level 1 metabolites)** | **Limitation** |
| --- | --- | --- | --- | --- | --- | --- | --- |
| Varma et al., 2018 [1] | AD/Control; AD progression across prodromal and preclinical stages | **Targeted** metabolomics; Platform: Biocrates AbsoluteIDQ p180 kit | Baltimore Longitudinal Study of Aging (BLSA); The Alzheimer’s Disease Neuroimaging Initiative (ADNI); Clinical sites in the United States and Canada. | BLSA: brain (autopsy) study sample+serum samples; ADNI: blood study sample | ~200 assayed | **Metabolic pathways:**Glycerophospholipid metabolism; Sphingolipid metabolism; Kennedy pathway; Land’s cycle; LCFA transport & ether lipid biosynthesis; Interconversion between PC and SM **level 1 metabolites:** Total N=26 **Sphingolipids:** SM C16:0, SM C16:1, SM C18:1, SM C24:1,SM C26:1,SM (OH) C14:1,SM (OH) C22:1,SM (OH) C22:2,SM (OH) C24:1 **Glycerophospholipids**: lysoPC a C17:0, lysoPC a C18:0, PC aa C38:4, PC aa C40:4, PC aa C40:5, PC aa C40:6, PC ae C34:0, PC ae C34:2, PC ae C36:0, PC ae C36:3, PC ae C36:4, PC ae C40:1, PC ae C42:3 **Amino acids**: Arginine biogenic amines: Serotonin, Spermidine Acylcarnitines: Propionylcarnitine | 1. Small number of brain tissue samples N=15 2.187 metabolites represent only a small proportion of the brain and blood metabolomes. 3. Selection bias of testing of pre-analytical variables in the BLSA serum samples |
| Mahajan el al., 2020 [2] | AD participants (N = 17): either AD or mild cognitive impairment (MCI); Asymptomatic AD (ASY) participants (N = 13): normal cognition within 1 year of death | **Targeted** metabolomics using capillary electrophoresis time-of-flight mass spectrometry (CE-TOFMS) | BLSA cohort | Cortical surface of autopsied brains from three regions: inferior temporal gyrus (ITG), middle frontal gyrus (MFG), and cerebellum (CB). | 27 focus out of 130 identified | **Pathways and level-1 metabolites**  Methionine cycle: betaine, choline, creatine, methionine (Met), methionine sulfoxide, S-adenosylmethionine (SAM), S-adenosylhomocysteine (SAH), and symmetric dimethylarginine (SDMA). Transsulfuration and glutathione synthesis: reduced glutathione (GSH), oxidized glutathione disulfide (GSSG), cysteine (Cys), and cystathionine. Polyamine synthesis and catabolism: putrescine and spermidine. Urea cycle: arginine, argininosuccinic acid, citrulline, N-acetylglutamate (NAG), ornithine, and urea. Glutamate-aspartate metabolism: alanine, aspartate, glutamine (Gln), and glutamate (Glu). Neurotransmitter metabolism: Gamma-amino-butyric acid (GABA) and N-acetylaspartate (NAA). | 1. Small proportion of all metabolites examined.  2. Cross-sectional nature of analyses.  3. Small sample size |
| Huo et al., 2020 [3] | AD pathology by histochemistry across 5 brain regions (e.g., counts of neuritic plaque burden, neurofibrillary tangles, and global AD pathology burden) and immunohistochemistry across 8 brain regions (e.g., β-amyloid load and PHFtau tangle density), semiquantitative measure of neurofibrillary tangle pathology (e.g., Braak stage), and neuritic plaque (e.g., CERAD score) | **Targeted** metabolomics, Biocrates AbsolutelDQ® p180 Kit (Biocrates Innsbruck, Austria) | ROS (Religious Orders Study) and MAP (Rush Memory and Aging Project): ROS started in 1994 and enrolls nuns priests and brothers from across the United States. MAP started in 1997 and enrolls lay persons from across northeastern Illinois. | brain tissue sample (from dorsolateral prefrontal cortex, N=111)+serum (N=530) | 154 serum metabolites+153 brain metabolites; 143 metabolites are present in both brain and blood samples | **level1 metabolites:** PC aa C30:0, Tetradecadienylcarnitine, Symmetric dimethylarginine, Threonine, Carnitine, Citrulline associated with various measures for AD neuropathology; acylcarnitine (tetradecadienylcarnitine [C14:2]) and three glycerophospholipids (PC aa C30:0, PC ae C34:0, PC ae C36:1) associated with both AD neuropathology and cognitive changes | 1. Focus on limited known metabolites 2. small sample size with both brain and blood samples  3. Only one brain region (dorsolateral prefrontal cortex) 4. Generalizibility |
| Batra et al., 2023 [4] | AD case: Braak stage ≥ 4 and CERAD score ≤ 2; Control case Braak stage ≤ 3 and CERAD score ≥ 3 | **Untargeted** metabolomics; Platform from Metabolon Inc using Ultrahigh Performance Liquid Chromatography-Tandem Mass Spectrometry (UPLC-MS/MS) | N=514 participants of Caucasian descent | Dorsolateral prefrontal cortex (DLPFC) | N=667 metabolites: lipids (42.7%), amino acids (22.6%), nucleotides (6.7%), carbohydrates (6.3%), cofactor and vitamins (4.3%), xenobiotics (3.7%), peptides (2.1%), and energy-related metabolites (1.5%) | **Pathways**: Cholesterol metabolism and steroid pathway; Neuroinflammation and oxidative stress; Osmoregulation;  **level1 metabolites:** **lipid:**1-stearoyl-2-arachidonoyl-GPE (18:0/20:4); 1-stearoyl-2-oleoyl-GPE (18:0/18:1); Glycerophosphoethanolamine (GPE); Glycerophosphoethanolamine; Glycerophosphorylcholine (GPC); **Amino acids**: Allo-threonine; N-acetylglutamate; N-acetylputrescine; N-acetyl-aspartyl-glutamate (NAAG); Carboxyethyl-GABA | 1. Cross-sectional study design 2. Diet and the gut microbiome uncontrolled confounders  3. Bulk tissue approach generates mixed metabolomic data from a variety of cell types and tissue compartments.  4. Postmortem tissue samples prone to biological and technical variation |
| Francois et al., 2022 [5] | AD/MCI/Control | **Untargeted** Metabolomics by gas chromatography-mass spectrometry (GC-MS) | N=80 from the South Australian Neurodegenerative Disease (SAND) cohort | Plasma samples | 489 untargeted metabolites | MCI Metabolite Biomarkers: N-Acetyl-alpha-D-glucosamine-1-phosphat, D-Mannose, Maleic acid, L-Norleucine, Myo-inositol, L-Glutamine, Creatinine-1, Isopentyl acetate, Itaconic acid **AD Metabolite Biomarkers:** Hypoxanthine, L-Glutamic acid, Epinephrine, 3-4-Dihydroxyphenylglycol, D-sedoheptulose-7-phosphate, N-acetyl-alpha-D-glucosamine-1-phosphate, Uridine, Methylmalonic acid, Erythrose-4-phosphate  **pathways:**arginine metabolism, alanine, aspartate and glutamate metabolism, pyruvate metabolism, pyrimidine metabolism, and purine metabolism. | N/A |
| Panyard et al., 2021 [6] | Six subgroups: (1) mild late-onset AD; (2) mild cognitive impairment (MCI); (3) age-matched healthy older controls (age>65); (4) middle-aged adults with a positive parental history of AD; (5) middle-aged adults with a negative parental history of AD; and (6) middle-aged adults with indeterminate parental history of AD | **Untargeted** metabolomics; Platform from Metabolon Inc using Ultrahigh Performance Liquid Chromatography-Tandem Mass Spectrometry (UPLC-MS/MS) | N=689 Wisconsin Alzheimer’s Disease Research Center (WADRC) and Wisconsin Registry for Alzheimer’s Prevention (WRAP) studies | Cerebrospinal fluid (CSF) | 412 metabolites, of which 354 were identified and 58 were of unknown structural identity. | **level1 metabolites** schizophrenia (N-delta-acetylornithine, alpha-tocopherol, ethylmalonate, N6-methyllysine, guanosine, malate, unknown metabolite X-24699, 2-hydroxy-3-methylvalerate),  cognitive performance (N-delta-acetylornithine, glutaroylcarnitine [C5], benzoate),  post-traumatic stress disorder (PTSD) (unknown metabolite X-24295) attention deficit hyperactivity disorder (ADHD)2(orotate and malate). | 1. small sample size; 2. Generalizability |
| Eteleeb et al., 2024 [7] | Early-AD, late-AD, presymptomatic, mild cognitive impairment (MCI), and control. | **Untargeted** metabolomics; Platform from Metabolon Inc using Ultrahigh Performance Liquid Chromatography-Tandem Mass Spectrometry (UPLC-MS/MS) | Knight Alzheimer Disease Research Center (Knight ADRC): 278 samples (255 AD and 23 control), non-hispanic white | Parietal cortical tissue samples. | 627 metabolites annotated | gamma-aminobutyrate (GABA) and choline Carbohydrate/sugar metabolites: glucose, mannitol, sorbitol, ribose, and UDP-glucose. Sphingolipid metabolites: sphingomyelin, sphingosine, and sphingadienine | 1. Mutiple omics data different brain regions and cohorts generated at **different times** on different samples using **different platforms.** |
| Novotny et al., 2023 [8] | AD diagnosis with variants in ADAD genes, carriers of TREM2 risk variants (TREM2), sporadic AD (sAD), neuropathological changes but no clinical symptoms (Presymptomatic), or no or minimal neuropathological AD lesions (CO). | **Untargeted** metabolomics; Platform from Metabolon Inc using Ultrahigh Performance Liquid Chromatography-Tandem Mass Spectrometry (UPLC-MS/MS) | Religious Orders Study and Memory and Aging Project (ROSMAP): 36 sAD and 55 CO serum samples, 233 sAD and 94 CO dorsolateral prefrontal cortex (DLPFC) | Serum and dorsolateral prefrontal cortex (DLPFC) | 627 metabolites | **16 level 1 metabolites:** Aspartate, 2. γ-Glutamylthreonine, 3. β-Citrylglutamate, 4. Glutamate, 5. N-Acetylglutamate, 6. Ergothioneine, 7. 3-Hydroxy-2-ethylpropionate, 8. 1,5-Anhydroglucitol (1,5-AG), 9. 2-Methylcitrate / Homocitrate, 10. Glutarate (C5-DC), 11. CDP-Choline, 12. CDP-Ethanolamine, 13. Glycerophosphoinositol, 14. Nicotinamide (Vitamin B3), 15. α-Tocopherol (Vitamin E), 16. Retinol (Vitamin A) The Super **Pathways** represented in the ADAD-associated metabolites: amino acids (48 metabolites), carbohydrates (12), cofactors and vitamins (9), energy (2), lipid (30), nucleotide (12), peptide (12), and xenobiotics (6).  Pathway analysis indicated overrepresented pathways in amino acid metabolism (including glutamate, glutathione, tryptophan, lysine, and histidine metabolisms), sphingolipid metabolism, and novel associations with vitamin pathways | 1. Small sample size 2. Lack of an age-matched control group |
| Batra et al., 2024 [9] | AD, progressive supranuclear palsy (PSP) and control | **Untargeted** metabolomics; Platform from Metabolon Inc using Ultrahigh Performance Liquid Chromatography-Tandem Mass Spectrometry (UPLC-MS/MS) | The Mayo Clinic Brain Bank: 142 AD, 156 PSP and 44 controls, non‐Hispanic Whites of North American or European descent. | cerebellar cortex (CER) and temporal cortex (TCX) regions | 658 metabolites from various metabolic “super‐pathways” , covering lipids (44.4%), amino acids (23.3%), nucleotides (7.0%), carbohydrates (5.3%), cofactors and vitamins (3.8%), peptides (2.7%), xenobiotics (2.7%), energy‐related metabolites (1.5%), and a series of uncharacterized metabolites (9.3%) | **Pathways:** urea cycle, alanine and aspartate, tyrosine, and branched‐chain amino acid (BCAA) metabolism, whereas glutathione metabolismpeptides, carbohydrates, energy metabolites, and nucleotide metabolism | 1. Unmeasured confounding BMI, years of education, and PMI, medication and supplement use, diet, the gut microbiome,and comorbidities. 2. Lacks a brain region substantially affected by neuropathology in PSP.  3. Less‐conservative false discovery rate threshold of 25% 4. Bulk tissue profiling lacks resolution at the single‐cell level |
| Milos et al., 2023 [10] | AD, MCI and healthy controls | **Untargeted** metabolomic using LC/MS and GC/MS | Recruited at the University Psychiatric Hospital Vrapce (Zagreb, Croatia): 40 healthy controls, 40 with MCI, and 40 patients with AD | Blood sampling (plasma) | GC–MS: 88 signals detected; LC-MS: 761 features detected in the positive mode and 783 in the negative ionization mode. | **Pathways**: minoacyl-tRNA biosynthesis, glyoxylate and dicarboxylate metabolism, glycine, serine and threonine metabolism, porphyrin and butanoate metabolism, citrate cycle, glutathione metabolism, alanine, aspartate and glutamate metabolism, and arginine and proline metabolism.  **Metabolites (not level 1):** glycine, serine, methionine, valine, threonine, proline, glutamic acid, citric acid, malic acid, glyceric acid, biliverdin, bilirubin, acetoacetate, succinic acid, and hydroxyproline. | 1. cross-sectional design  2. Uncontrolled confounding |
| Peña-Bautista, Roca, López-Cuevas, et al., 2019 [11] | Early AD patients and healthy controls. | **Untargeted** metabolomic using ultra-performance liquid chromatography coupled to time-of-flight mass spectrometry (UPLC-Q-ToF MS) | Neurology Unit of the University and Polytechnic Hospital La Fe, Valencia (Spain): 29 with early AD and 29 with healthy controls | Blood samples | Up to thousands | **level 1 metabolites:** alpha-d-galactosyl undecaprenyl diphosphate; LysoPC(18:1); LysoPC(P-18:0); LysoPE(0:0/22:1(13Z); LysoPE(22:1(13Z)/0:0); CL(8:0/14:0/18:2(9Z,11Z)/18:2(9Z,11Z) CL(8:0/i14:0/18:2(9Z,11Z)/18:2(9Z,11Z)) | 1. Small sample size |
| Peña-Bautista, Roca, Hervás, et al., 2019 [12] | Mild Cognitive Impairment due to Alzheimer's disease patients (MCI-AD) and healthy controls | **Untargeted** metabolomic using ultra-performance liquid chromatography coupled to time-of-flight mass spectrometry (UPLC-Q-ToF MS) | Neurology Unit (University and Polytechnic Hospital La Fe, Valencia (Spain): 29 with MCI-AD and 29 with healthy controls | Plasma samples | Up to thousands | **Metabolites (level 1/2/3):** choline, carnitine and nicotinamide derivatives, depsides, tocopherols, dipeptides, Lyso PEs, inositol derivatives. | N/A |
| Hajjar et al., 2020 [13] | MCI and normal controls | **Untargeted** metabolomics using LC-MS | Brain Stress Hypertension and Aging program (B‐SHARP) at Emory University, N=185 | CSF samples | 13,064 features were detected, and 8043 features met the data filtering criteria and used for downstream analyses. | **level 1 metabolites:** L‐Ribulose, D‐Sorbitol, Maltose, S‐Adenosylhomocysteine, 7,8‐Dihydroneopterin 3′triphosphate, Galactosylglycerol, N‐Acetyl‐D‐glucosamine 6‐phosphate, Salsolinol 1‐carboxylate, N‐acetyl‐alpha‐D‐glucosamine, S‐Adenosylmethionine, beta‐D‐Galactosyl‐1,4‐N‐acetyl‐D‐glucosamine, Vanillylmandelic acid, Levothyroxine, 3‐beta‐D‐Galactosyl‐sn‐glycerol, GlcNAc **pathways:** N-Glycan Degradation, Sialic acid metabolism, Aminosugars metabolism, Galactose metabolism, Methionine and cysteine metabolism, Vitamin B12 (cyanocobalamin) metabolism, Urea cycle/amino group metabolism, Keratan sulfate degradation, Tyrosine metabolism, Biopterin metabolism, Hyaluronan metabolism, Purine metabolism, Selenoamino acid metabolism | 1. Cross‐sectional design 2. Number of identified features that could not be matched to known metabolites or matched to multiple metabolites |
| Shao et al., 2020 [14] | AD, MCI, healthy controls and non-AD neurological disease controls | **Untargeted** metabolomics using UPLC (Waters Corporation, Manchester, UK) coupled to a tripleTOF™5600 plus (Applied Biosystems, Foster City, CA) mass spectrometry system. | First Affiliated Hospital of Dalian Medical University, China, N=138 | plasma samples | 2,412 ion signatures in ESI+ mode and 2,672 ion signatures in ESI- mode | **level1 metabolites**: cholic acid, chenodeoxycholic acid, allocholic acid, indolelactic acid and tryptophan; **pathways:** alpha linolenic acid and linoleic acid metabolism, bile acid biosynthesis, caffeine metabolism, thyroid hormone synthesis, and catecholamine biosynthesis | the samples size is insufficient |


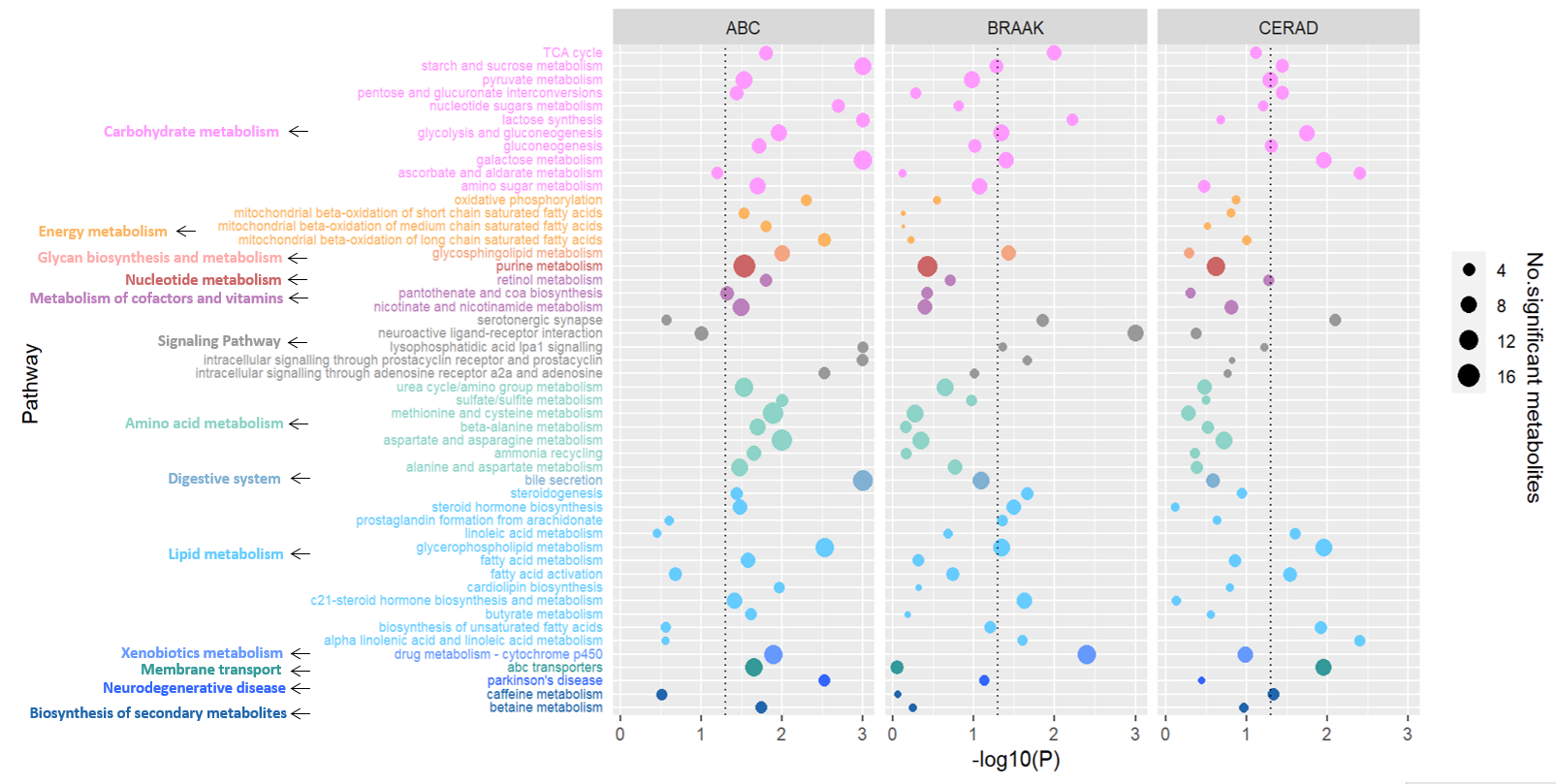


**Figure S1.** Bubble plots of enrichment pathways associated with three Alzheimer's disease neuropathology using multivariable linear regression. Size of bubble represents the number of significant putative metabolites (p-value< 0.05) with m/z matched among each metabolic pathway using *metapone*. X-axis represents the negative log10 of p-value of the association between each significant pathway and outcomes. Color of both bubble and y-axis label represents that class of each metabolic pathways. The dashed line represents the threshold of p-value at 0.05.

Abbreviations: ABC: NIA-AA Alzheimer's disease neuropathologic change (ADNC); BRAAK: Braak stage for neurofibrillary degeneration; CERAD: CERAD score for density of neocortical neuritic plaque.


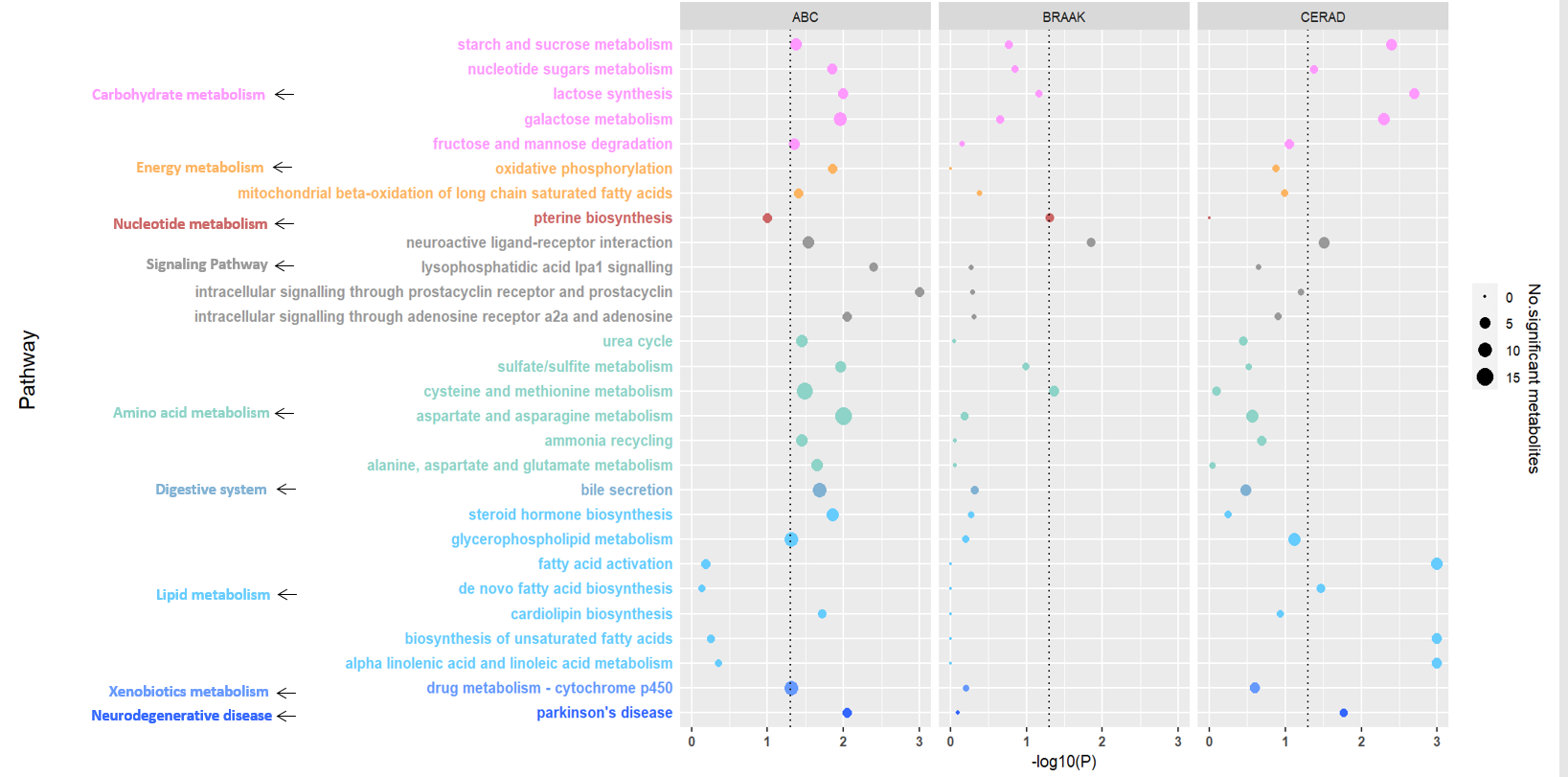


**Figure S2.** Bubble plots of enrichment pathways associated with three Alzheimer's disease neuropathology using ordinal logistic regression additionally adjusted for cell type. Size of bubble represents the number of significant putative metabolites (p-value< 0.05) with m/z matched among each metabolic pathway using *metapone*. X-axis represents the negative log10 of p-value of the association between each significant pathway and outcomes. Color of both bubble and y-axis label represents that class of each metabolic pathways. The dashed line represents the threshold of p-value at 0.05.

Abbreviations: ABC: NIA-AA Alzheimer's disease neuropathologic change (ADNC); BRAAK: Braak stage for neurofibrillary degeneration; CERAD: CERAD score for density of neocortical neuritic plaque.


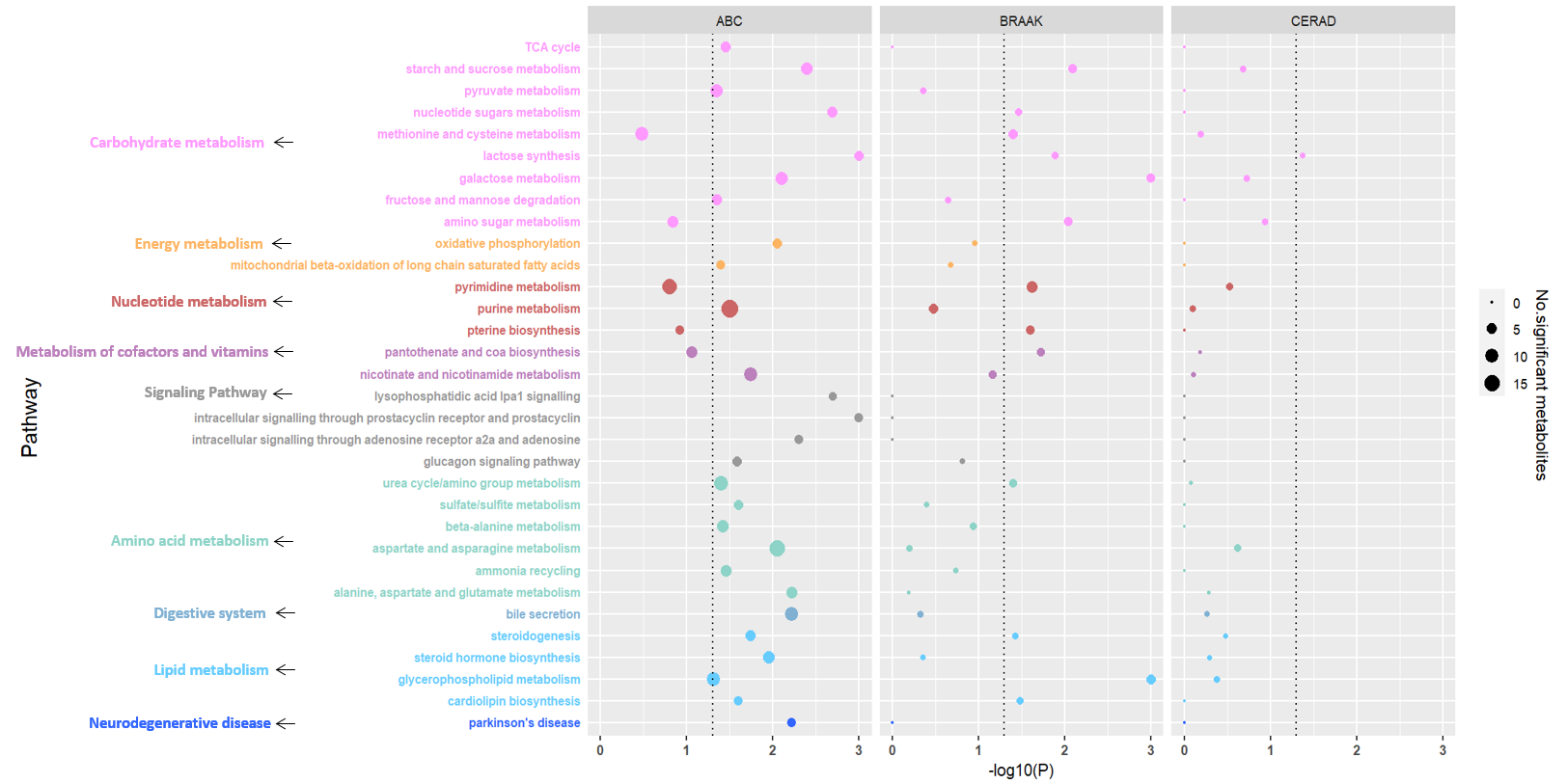


**Figure S3.** Bubble plots of enrichment pathways associated with three Alzheimer's disease neuropathology using ordinal logistic regression additionally adjusted for principal component. Size of bubble represents the number of significant putative metabolites (p-value< 0.05) with m/z matched among each metabolic pathway using *metapone*. X-axis represents the negative log10 of p-value of the association between each significant pathway and outcomes. Color of both bubble and y-axis label represents that class of each metabolic pathways. The dashed line represents the threshold of p-value at 0.05.

Abbreviations: ABC: NIA-AA Alzheimer's disease neuropathologic change (ADNC); BRAAK: Braak stage for neurofibrillary degeneration; CERAD: CERAD score for density of neocortical neuritic plaque.

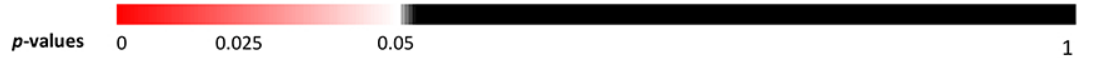


**Figure S4.** Heat map of p-values. Each cell was colored by the p-value of the association of each metabolic pathway and AD neuropathology at raw p-value 0.05 and FDR q-value 0.2 as cut-off input.

Abbreviations: ABC: NIA-AA Alzheimer's disease neuropathologic change (ADNC); BRAAK: Braak stage for neurofibrillary degeneration; CERAD: CERAD score for density of neocortical neuritic plaque.

**References:**

1. Varma, V.R., et al., *Brain and blood metabolite signatures of pathology and progression in Alzheimer disease: A targeted metabolomics study.* PLoS medicine, 2018. **15**(1): p. e1002482.

2. Mahajan, U.V., et al., *Dysregulation of multiple metabolic networks related to brain transmethylation and polyamine pathways in Alzheimer disease: A targeted metabolomic and transcriptomic study.* PLoS medicine, 2020. **17**(1): p. e1003012.

3. Huo, Z., et al., *Brain and blood metabolome for Alzheimer's dementia: findings from a targeted metabolomics analysis.* Neurobiology of aging, 2020. **86**: p. 123-133.

4. Batra, R., et al., *The landscape of metabolic brain alterations in Alzheimer's disease.* Alzheimer's & Dementia, 2023. **19**(3): p. 980-998.

5. François, M., et al., *Multi-omics, an integrated approach to identify novel blood biomarkers of Alzheimer’s disease.* Metabolites, 2022. **12**(10): p. 949.

6. Panyard, D.J., et al., *Cerebrospinal fluid metabolomics identifies 19 brain-related phenotype associations.* Communications biology, 2021. **4**(1): p. 63.

7. Eteleeb, A.M., et al., *Brain high-throughput multi-omics data reveal molecular heterogeneity in Alzheimer’s disease.* PLoS biology, 2024. **22**(4): p. e3002607.

8. Novotny, B.C., et al., *Metabolomic and lipidomic signatures in autosomal dominant and late‐onset Alzheimer's disease brains.* Alzheimer's & Dementia, 2023. **19**(5): p. 1785-1799.

9. Batra, R., et al., *Comparative brain metabolomics reveals shared and distinct metabolic alterations in Alzheimer's disease and progressive supranuclear palsy.* Alzheimer's & Dementia, 2024. **20**(12): p. 8294-8307.

10. Milos, T., et al., *Metabolic profiling of Alzheimer's disease: untargeted metabolomics analysis of plasma samples.* Progress in Neuro-Psychopharmacology and Biological Psychiatry, 2023. **127**: p. 110830.

11. Peña-Bautista, C., et al., *Metabolomics study to identify plasma biomarkers in alzheimer disease: ApoE genotype effect.* Journal of Pharmaceutical and Biomedical Analysis, 2019. **180**: p. 113088-113088.

12. Peña-Bautista, C., et al., *Plasma metabolomics in early Alzheimer's disease patients diagnosed with amyloid biomarker.* Journal of proteomics, 2019. **200**: p. 144-152.

13. Hajjar, I., et al., *Untargeted metabolomics reveal dysregulations in sugar, methionine, and tyrosine pathways in the prodromal state of AD.* Alzheimer's & Dementia: Diagnosis, Assessment & Disease Monitoring, 2020. **12**(1): p. e12064.

14. Shao, Y., et al., *Alteration of metabolic profile and potential biomarkers in the plasma of Alzheimer’s disease.* Aging and disease, 2020. **11**(6): p. 1459.
